# Targeting BRD4 in gastric cancer: promoting apoptosis and suppressing tumor progression

**DOI:** 10.3389/fphar.2026.1835830

**Published:** 2026-06-24

**Authors:** Xu-Liang Liao, Xiao-Hai Song, Hao Cai, Lu Niu, Yonghong Mao, Yue Pan, Ai Lin, Tingting He, Guangzhi Ma, XiuLi Zheng, Gang Yuan

**Affiliations:** 1 Department of Thoracic Surgery and Institute of Thoracic Oncology, Frontiers Science Center for Disease-Related Molecular Network, West China Hospital, Sichuan University, Chengdu, China; 2 Division of Gastrointestinal Surgery, Department of General Surgery, West China Hospital of Sichuan University, Chengdu, China; 3 Laboratory of Precision Therapeutics, West China Hospital, Sichuan University, Chengdu, China

**Keywords:** biomarker, BRD4 degrader ARV771, gastric cancer, targeted therapy, tumor progression

## Abstract

**Background:**

The MYC pathway is highly activated in gastric cancer (GC), but the MYC oncogene’s structural biology makes direct pharmacological inhibition very difficult. We hypothesized that BRD4, as a major co-activator of MYC, maintains MYC-dependent survival circuits, thus making BRD4 a key therapeutic vulnerability in late GC.

**Methods:**

We carried out a comprehensive transcriptomic analysis by utilizing large-scale data sets from The Cancer Genome Atlas (TCGA) and Gene Expression Omnibus (GEO), then these computational forecasts were later verified in strict clinical cohorts via molecular and histological methods. Functional verification was done through genetic and pharmacological interference by means of CRISPR-Cas9 gene deletion and PROTAC-induced protein breakdown with ARV771, ultimately, the therapeutic efficacy and systemic safety of these interventions were assessed *in vivo* with mouse xenograft models.

**Results:**

Our analyses revealed that the expression of BRD4 was significantly increased in GC tissues and there was a strong connection between this increase and the MYC hyperactivation, advanced TNM staging, and shortened patient survival. Our knockout (KO) experiments found that the viability of tumor cells depended greatly on the BRD4 - MYC - BCL2 signaling pathway because when BRD4 was depleted, the expression of BCL2, a crucial anti-apoptotic protein, decreased substantially. When pharmacological intervention was applied using ARV771, these cancer-related characteristics were effectively reversed, resulting in the suppression of tumor proliferation, the decrease of BCL2 levels, and significant inhibition of the growth of xenograft tumors *in vivo* without observable toxicity.

**Conclusion:**

Our findings suggest that BRD4 acts a chief epigenetic controller which promotes the progress of GC by intensifying MYC-dependent survival and ARV771 blocks this survival advantage, thus presenting a highly compelling rationale for integrating BRD4 degraders into modern targeted regimens.

## Introduction

Gastric cancer poses a substantial global health problem as it ranks third among the causes of cancer - related deaths across the world and there is an unequal occurrence of this disease in Asian populations, although the global incidence rate has remained stable over the past few decades, the total number of new cases and deaths keeps on rising because of demographic aging and changes in population trends (Zhang et al., 2024), recently, epidemiological data showed a worrying rise in the incidence of early-onset gastric cancer, especially among young Hispanic men which suggests that our current knowledge about its causative factors is not comprehensive (Wang et al., 2018; Merchant et al., 2017), *Helicobacter pylori* infection has long been recognized as a major risk factor but the development of gastric cancer is multi-factorial as a complicated interaction exists between genetic predisposition, dietary carcinogens, and lifestyle elements that all contribute to promoting malignant conversion (Lin et al., 2021; Shin et al., 2023; Huang et al., 2024) and the continuous and increasing disease load emphasizes the urgent necessity to find new, practical therapeutic targets in the gastric tumor microenvironment.

The contemporary management of gastric cancer relies on a multidisciplinary strategy and in the past, for early-stage cases, surgery was the primary treatment method with D2 lymph node removal being a specific example (Chen et al., 2021; Meyer and Wilke, 2011), but for patients with locally advanced or metastasized cancer, surgery alone didn’t offer much in terms of survival benefits (Kang and Kim, 2021), so cytotoxic chemotherapy using combinations of fluoropyrimidine and platinum became the main form of palliative care, yet a major issue in clinical practice was that resistance to chemotherapy developed quickly which kept the average survival time around 12 months and also brought about substantial side effects related to treatment (Wagner et al., 2017; Kim, 2005).

The inherent limitations of traditional chemotherapy have spurred a shift in oncology towards the growth of precision medicine and the clinical verification of trastuzumab for tumors with HER2-amplification provided evidence for targeted treatment methods (Yoong et al., 2011; Selim et al., 2019; Kahraman and Yalcin, 2021), but the clinical use of this method was restricted because HER2-amplification made up only a small part of the molecular types in the larger group of gastric cancer patients (Yuan et al., 2016). New therapeutic drugs aimed at newly-found antigens like CLDN18.2 and BTK were being researched at that time and had shown initial effectiveness in early-stage clinical trials which called for more research (Singh et al., 2017; Portillo-Mino et al., 2025), however the great spatial and temporal differences in gastric tumors usually held back the long-lasting clinical results that could be achieved with single-drug targeted treatments (Kumar et al., 2018; Patel and Cecchini, 2020). At the same time, although the arrival of immune checkpoint blockade, like PD1/PD-L1 inhibitors, marked an important progress in treatment for certain molecular groups-namely, tumors that were microsatellite-instability-high (MSI - H) or Epstein-Barr virus (EBV)-positive-most patients got little clinical benefit because this poor effectiveness was due to inborn resistant mechanisms and not enough reliable predictive biomarkers to find out who would respond (Kono et al., 2020; Hou et al., 2023).

The development of a therapeutic standstill has led to a turn towards making use of high throughput genomic data so as to clarify the basic disease biology, large - scale sequencing projects have found characteristic mutational marks in important genes like TP53, ARID1A, and CDH1 (Cai et al., 2019; Ali et al., 2015), also the combination of bulk and single - cell transcriptomics has made it possible to draw out complicated signaling pathways that set malignant gastric cells apart from benign ones (Wang et al., 2024; Ma et al., 2025), by analyzing these public data repositories thoroughly, our research found a constant abnormality which was the widespread and strong overactivity of the MYC signaling pathway in all gastric tumor tissues.

The MYC protein, a long known oncogenic driving force, has traditionally been regarded as being resistant to drug treatment because it lacks substantial hydrophobic cavities, a feature that has hindered direct pharmaceutical interference, so research has veered towards targeting the epigenetic apparatus that sustains MYC induced transcriptional mis-regulation and among these targets, BRD4 (Bromodomain containing protein 4), a crucial transcriptional co-activator of MYC. BRD4 is known to be significantly elevated in several types of cancer, including hepatocellular carcinoma and breast cancer, where it acts as a major driver of disease progression. Similarly, BRD4 is constantly overproduced in gastric tumors, thus we hypothesized that this BRD4 overproduction was not just a coincidental occurrence but signified a basic dependence that spurred unrestrained cellular growth. However, it is important to note that epigenetic regulators like BRD4 are also crucial in healthy cell physiology, which makes it a potentially challenging therapeutic target. The novelty of this study lies in systematically uncovering the specific BRD4-MYC-BCL2 survival dependency in gastric cancer and being the first to demonstrate that the PROTAC degrader ARV771 can safely and potently dismantle this oncogenic network *in vivo*. Consequently, a vital question still existed as to whether the therapeutic breakdown of BRD4 could efficiently and securely disrupt the disease-causing mechanisms in gastric cancer without causing severe systemic toxicity, which was the main emphasis of this study.

## Materials and methods

### Acquisition and processing of TCGA gastric cancer transcriptomic data

To thoroughly clarify the molecular disruptions in gastric cancer (GC), a strong analysis of large - scale cohort data is necessary so we got transcriptional profiles in HTSeq - FPKM form and matching clinical annotations for 375 GC samples and 32 paired adjacent normal samples from The Cancer Genome Atlas (TCGA) and to reduce possible biases from sequencing depth when comparing different samples, all expression matrices were made standard by converting them into transcripts per million (TPM) and this normalized dataset was used for differential expression analysis with the limma R package which uses linear modeling and empirical Bayes moderation to make variance estimates smaller thus increasing statistical dependability especially in datasets that have built - in clinical differences (Smyth, 2004).

To explore the biological significance of these transcriptomic changes, we carried out a pathway - level disturbance analysis by means of Gene Set Enrichment Analysis (GSEA) in the clusterProfiler package and this analysis made use of gene set groups from the Molecular Signatures Database (MSigDB) (Subramanian et al., 2005; Mootha et al., 2003; Yu et al., 2012), and pathways were regarded as being considerably overactive in the tumor situation when they satisfied the statistical standards of a normalized enrichment score (NES) greater than one and an adjusted P-value less than 0.05, and to especially look into the function of epigenetic regulation, we built a special gene set, namely, the BRD4 Pathway (BioGRID), containing experimentally confirmed BRD4 - interacting proteins, and the enrichment of this customized gene set was assessed within the gastric cancer (GC) group through the existing GSEA framework, and to clarify the regulatory connections among these highlighted genes, we constructed a Pearson correlation network which was shown visually with the ggplot2, ggpubr, and ggExtra packages in R.

### Retrieval and preprocessing of GEO transcriptomic datasets

To reduce the risk of artifacts specific to a particular group, we cross - validated our findings from TCGA by using an independent group of datasets from the Gene Expression Omnibus (GEO), which consisted of bulk RNA-seq data including several groups of gastric cancer (GC) and non - tumor tissues (such as GSE19826, GSE112369, GSE65801, GSE51575, GSE13195, GSE33335, GSE63089, and GSE103236) and also incorporated controlled *in vitro* transcriptomic profiles like wild - type compared to BRD4 - knockout HGC27 cells (GSE134150) and DMSO vs. ARV771 treated lymphoma models (RIVA and U2932, GSE119241) to assess the transcriptional signs of pharmacological and genetic BRD4 removal, and during the data preparation process, platform - specific annotation files were used to match probe - level signals to official gene names and for genes represented by multiple probes, the average expression value was computed and since these repositories offered pre - normalized data matrices, no additional normalization was carried out.

Bulk sequencing offers a broad view of tumor traits but the microenvironment of gastric tumors shows great diversity, so in order to figure out exactly where BRD4 is active within cells, we examined the GSE134520 single - cell RNA - sequencing dataset which had 32,332 single cells and on average each cell had 1,941 genes detected, and the single - cell data came from the TISCH database as raw.h5 files then it was strictly controlled for quality and processed by the MAESTRO and Seurat computational tools after that the dimension was reduced with the t - SNE algorithm to build a map showing the relative BRD4 expression among different cell groups.

### BRD4-associated pathway enrichment analysis in gastric cancer

To explore the functional consequences of BRD4 misregulation, we divided patient groups from four separate GEO datasets (GSE19826, GSE112369, GSE51575, and GSE65801) into two subgroups, namely, BRD4 - high and BRD4 - low, based on median expression levels as the dividing line and then utilized the empirical Bayes approach within the limma package (Smyth, 2004) to identify differentially expressed genes (DEGs) which distinguished these divisions with statistical significance set at a |log2 fold change| greater than 0 and an adjusted p - value less than 0.05.

To explore the biological meaning of the differentially expressed genes (DEGs), we carried out functional annotation through Gene Ontology (GO) and Kyoto Encyclopedia of Genes and Genomes (KEGG) pathway analyses (Ashburner et al., 2000; Ogata et al., 1999) which individually mapped the sets of up - regulated and down - regulated genes, and to figure out if these single - gene alterations signified coordinated shifts in biological pathways, we performed a Gene Set Enrichment Analysis (GSEA) taking the Molecular Signatures Database (MSigDB) as a reference (Subramanian et al., 2005; Mootha et al., 2003; Yu et al., 2012), and to reduce the likelihood of short - term false positives usually related to single - cohort studies, we recognized highly conserved regulatory targets by calculating the intersections of DEGs and enriched pathways across several independent datasets via an online Venn diagram platform (Tang et al., 2023), and all analysis steps and visualizations were done using a set of R packages such as clusterProfiler, ggplot2, enrichplot, pathview, ggpubr, ggExtra, circlize, corrplot, pheatmap, colorspace, stringi, DOSE, BiocManager, and org.Hs.eg.db.

### Integrative mapping of the downstream MYC axis

Given the established functional crosstalk between BRD4 and the MYC oncogenic network, we sought to systematically define direct downstream MYC targets. This required the integration of multiple bioinformatics tiers: established regulatory interactions were aggregated from TRRUST (https://www.grnpedia.org/trrust/), KnockTF (http://www.licpathway.net/KnockTF/index.html), ChEA (Welch et al., 2014), GTRD (http://gtrd.biouml.org), and ENCODE (www.encodeproject.org) databases. To anchor these predictions in physical binding evidence, public ChIP-seq tracks were audited via ChIP-Atlas (https://chip-atlas.org/). Finally, sequence-level motif scanning was executed using the PWMEnrich package and the FIMO tool (JASPAR database) (http://jaspar.genereg.net/downloads/ and http://meme-suite.org/tools/fimo), allowing us to distill a high-confidence pool of direct, structurally validated MYC targets.

### Clinicopathological correlation and survival analysis in gastric cancer patients

To convert molecular signatures into clinically relevant prognoses, corresponding clinical factors such as age, gender, and TNM staging were retrieved from the TCGA database and the ideal prognostic threshold for BRD4 expression was established by means of the survival package (R version 4.4.1), but since age and an advanced TNM stage had inherent biases in retrospective survival studies (Sano et al., 2016; Woo et al., 2016; Li et al., 2019), a strict Propensity Score Matching (PSM) procedure was carried out to separate the independent prognostic effect of BRD4, and with the help of the matching package (Mokdad et al., 2018), propensity scores were computed through logistic regression considering age, gender, and stage which made it possible to match BRD4 - high and BRD4 - low patients at a ratio of 1:1 (with a caliper of 0.05), then after matching, the overall survival (OS) was evaluated by Kaplan - Meier curves and log - rank tests and additional clinical validations like *H. pylori* status and tumor differentiation were conducted in the GSE13195 and GSE103236 cohorts using independent - samples t - tests and chi - square tests respectively.

### Histopathological validation via immunohistochemistry

A total of 64 gastric carcinoma (GC) specimens and 8 paired adjacent non - tumor samples were obtained from the Department of Pathology, West China Hospital, Sichuan University (approval number: 2025(1275)), and the tissue samples were fixed in 10% neutral buffered formalin for no less than 2 weeks before being dehydrated and embedded in paraffin, and immunohistochemical (IHC) staining was carried out in accordance with the existing protocols (Cao et al., 2021) with some slight changes, and briefly speaking, after deparaffinization, antigen retrieval was done in 10 mM sodium citrate buffer (pH 6.0) by using a pressure cooker at 125 °C for 5 minutes, and the sections were then soaked overnight at 4 °C in a rabbit polyclonal anti-BRD4 antibody (Proteintech, cat#28486; dilution 1:400), and chromogenic development and slide mounting were performed through standard procedures, and whole - slide digital images were captured with Olyvia software (Olympus).

Two pathologists independently scored staining in a blinded manner using the Immunoreactive Score (IRS) system, which combines staining intensity (0: negative; 1: weak; 2: moderate; 3: strong) and percentage of positive cells (0: 0%; 1: 1%–25%; 2: 26%–50%; 3: 51%–75%; 4: 76%–100%). IRS scores (range 0–12) were calculated as the product of these two parameters. BRD4 expression was categorized as high or low based on IRS values. Differences in clinicopathological features were analyzed using chi-square or Fisher’s exact test for categorical variables and two-tailed t-tests for continuous variables.

### Cell lines and culture conditions

Human gastric cancer (GC) cell lines such as AGS (RRID: CL-0022), HGC27 (RRID: CL-0107), MKN45 (RRID: CL-0292), and MKN74 (RRID: CL-0730) were obtained from Procell Life Science and Technology (Wuhan, China), the AGS, MKN45, and MKN74 cell lines were grown in RPMI-1640 medium (Gibco, United States) while HGC27 cells were kept in DMEM (Gibco, United States), both media had been added with 10% fetal bovine serum (PAN, Germany) and 50 U/mL penicillin-streptomycin (Gibco, United States), all cell cultures were incubated at 37 °C in a moist atmosphere with 5% CO_2_, short tandem repeat (STR) profiling was carried out within the past 3 years to verify all cell lines and it was confirmed that there was no *mycoplasma* contamination in them.

### Plasmid construction and lentiviral transduction

BRD4 - knockout cell lines were created by inserting single guide RNAs (sgRNAs) that targeted BRD4 (sgBRD4-1: 5′-GGG​AAC​AAT​AAA​GAA​GCG​CT-3’; sgBRD4-2: 5′- ACA​GGA​GGA​GGA​TTC​GGC​TG-3′) into the lentiCRISPRv2 vector which had been made linear with BsmBI (NEB #B7024), for lentivirus generation, HEK293T cells were co - transfected with the transfer plasmids and packaging plasmids (pMD2.G and psPAX2) via polyethylenimine (PEI; YEASEN, 40816ES01), after that, the viral supernatants were collected, strained, and used to infect the target gastric cancer (GC) cell lines.

### Western blot analysis

Cells in the logarithmic growth phase were lysed in RIPA buffer (Beyotime, P0013B) supplemented with protease inhibitors on ice for 30 min, followed by ultrasonication at 50 W for 5 min. Lysates were centrifuged at 12,000 rpm, 4 °C, 15 min, and supernatants were collected. Protein concentrations were quantified using a BCA assay kit (Beyotime, P0012). Equal amounts of protein (20–40 μg) were denatured in 5× SDS loading buffer at 95 °C for 5 min, separated by 10%–12% SDS-PAGE, and transferred to PVDF or NC membranes (300 mA, ice bath, 60–90 min). Membranes were blocked with 5% non-fat milk in PBST, incubated overnight at 4 °C with primary antibodies (anti-BRD4, β-actin, BCL-2), followed by HRP-conjugated secondary antibodies (1:10,000). Signals were detected using ECL chemiluminescent substrate (4A BIOTECH, 4AW012-200). We utilized Image Lab 6.1 software to extract the densitometric values for all presented WB bands, and applied the rank-sum test to rigorously assess statistical significance. The complete quantitative readouts, alongside their corresponding statistical outcomes are presented in the ‘Quantification of WB bands’ sheet of the extend excel file.

### CCK-8 assay for cell proliferation and cytotoxicity

Cell proliferation was assessed using the CCK-8 kit (YEASEN, 40203ES60). Log-phase cells (sgCtrl, sgBRD4-1, sgBRD4-2) were seeded in 96-well plates (1–2 × 10^3 cells/well, six replicates), and proliferation was measured at indicated time points using absorbance at 450 nm. For IC50 determination, cells were treated with ARV771 (0.0001–100 nM) or DMSO for 48 h, and viability curves were plotted. Repeated-measures ANOVA was used for statistical comparison.

### Colony formation assay

AGS and HGC27 cells (600 per well) and MKN45 cells (3,000 per well) in the logarithmic growth phase were seeded into 6 well plates, then they were cultured for 12–14 days with the culture medium being refreshed every other day, after that the cells were fixed by 4% paraformaldehyde and dyed with 0.1% crystal violet, colonies larger than 50 μm in diameter were counted and all experiments were carried out three times and statistical analysis was done through two-tailed t-tests.

### Animal experiments and *in vivo* drug evaluation

All animal experiments were carried out in line with the ethical principles approved by the Medical Ethics Committee of West China Hospital (approval number: 20240116001), female BALB/c nude mice which were 8 week old were kept under specific pathogen free (SPF) circumstances, tumors were set up through subcutaneous injection of 2 × 10^6 MKN45 cells, when tumor volumes reached around 50 mm^3^, the mice were randomly divided into either the vehicle control group or the ARV771 treatment group (10 mg/kg, given intravenously every 2 days), the ARV771 substance was made in a solvent combination consisting of 10% dimethyl sulfoxide (DMSO), 40% polyethylene glycol 300 (PEG300), 5% Tween-80% and 45% physiological saline, tumor sizes were measured every 3 days and tumor volumes were calculated using formula L x W^2^, at the end of the experiment, main organs were collected for histopathological examination by hematoxylin and eosin (H&E) staining, tumor load and body weight figures are shown as mean ± standard deviation (SD).

### Hematoxylin and eosin staining

After being fixed in 10% neutral buffered formalin, the tissue samples were prepared for paraffin embedding, cut into sections 5 μm thick, and then dyed with hematoxylin and eosin (H&E), and a thorough histopathological inspection of the heart, liver, spleen, lung, and kidney was carried out to assess the possible toxicological impacts of ARV771.

### Statistical analysis

All experimental procedures were carried out in no less than three separate repetitions if not otherwise stated, data were shown as the average ±standard error (SE), statistical importance was decided through two - sided tests and a p - value of less than 0.05 was regarded as statistically important, and data analysis and visualization were done by means of GraphPad Prism version 8 and R statistical software (version 4.4.1).

## Results

### BRD4 hijacks the MYC pathway to fuel gastric cancer progression

**FIGURE 1:**
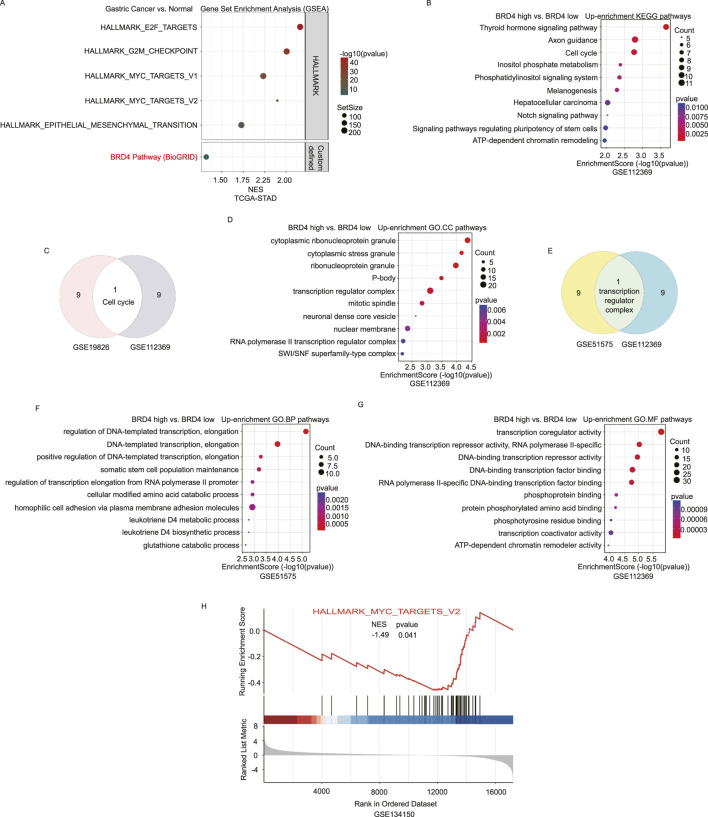
The BRD4 and MYC signaling pathways are hyperactivated, and MYC pathway activation is more pronounced in gastric cancer tissues with higher BRD4 levels. **(A)** The top five significantly upregulated pathways in gastric cancer (GC) tissues compared to adjacent normal tissues, along with the significant upregulation of our custom-defined BRD4 pathway; **(B)** The top ten significantly upregulated KEGG pathways in BRD4-high GC tissues from the GSE112369 dataset; **(C)** Venn diagram showing that the cell cycle is the commonly upregulated KEGG pathway in BRD4-high GC tissues across the GSE19826 and GSE112369 datasets; **(D)** The top ten significantly upregulated Gene Ontology Cellular Component (GO-CC) terms in BRD4-high GC tissues from the GSE112369 dataset; **(E)** Venn diagram indicating that the transcription regulator complex is the commonly upregulated GO-CC term in BRD4-high GC tissues across the GSE51575 and GSE112369 datasets; **(F)** The top ten significantly upregulated Gene Ontology Biological Process (GO-BP) terms in BRD4-high GC tissues from the GSE51575 dataset; **(G)** The top ten significantly upregulated Gene Ontology Molecular Function (GO-MF) terms in BRD4-high GC tissues from the GSE112369 dataset; **(H)** The Hallmark GSEA from GSE134150 confirmed that after CRISPR-mediated BRD4 ablation in GC cells, MYC signaling was significantly weakened. Statistical method: Hypergeometric test **(B,D,F,G)**; Empirical permutation test **(A,H)**. (Abbreviations: T, tumor tissue; A, adjacent normal tissue; KEGG, Kyoto Encyclopedia of Genes and Genomes; GSEA, Gene Set Enrichment Analysis; CRISPR, Clustered Regularly Interspaced Short Palindromic Repeats).

To find actionable weaknesses in gastric cancer, attention should be paid to overactive signaling systems. Driven by this rationale, when analyzing data from The Cancer Genome Atlas (TCGA) using Gene Set Enrichment Analysis (GSEA), a clear molecular mark was found. This mark showed that in malignant tissues compared to nearby healthy mucosa, there was a great deal of activity in pathways controlling the G2/M checkpoint, the process of epithelial - mesenchymal transition (EMT), E2F target genes, and especially MYC target genes (V1 and V2) (Figure 1A; Supplementary Figure S1A). Since it was known that MYC could not be targeted with drugs due to its intrinsically disordered protein topology and the notorious absence of traditional hydrophobic binding pockets, our research turned to BRD4, which plays a crucial role in regulating MYC at the epigenetic level. To validate whether BRD4 is indeed the upstream driver in our context, by using a special set of genes related to BRD4 taken from BioGRID, it was shown that this particular network of genes involved in transcription was highly active in tumors (Figure 1A; Supplementary Figure S1B). This molecular footprint prompted us to conceptualize a model where upstream chromatin readers serve as the actual gatekeepers of this intractable oncogenic signaling.

Having established the potential role of BRD4, To further explore this, multiple independent GEO cohorts (GSE19826 and GSE112369) were further analyzed, and it was shown that tissues divided by high BRD4 expression always had an overly active cell cycle progression and the activation of many cancer - promoting pathways (Figures 1B,C; Supplementary Figure S1C). When looking into the function of BRD4 via Gene Ontology mapping, it was found that places where BRD4 is highly expressed are full of things related to RNA polymerase II - dependent transcriptional elongation and co - activation (Figures 1D–G; Supplementary Figure S1D). BRD4 binds to promoter areas and strongly boosts oncogenic transcription; therefore, to see how this boost works within the MYC axis, a tissue - level correlation study was done and it was discovered that stomach cancer samples with high BRD4 levels had a great increase in the MYC pathway (Supplementary Figure S1E). Moving from connection to cause, this link was tested experimentally and knocking out BRD4 in cultured stomach cancer cells led to a big reduction in downstream MYC signaling (Figure 1H), thus all these findings proved that BRD4 is not just a quiet observer in epigenetics but an active force driving the MYC oncogenic process. By tethering the enhancer landscapes to the transcriptional machinery, BRD4 essentially acts as the master rheostat for MYC dependency.

### BRD4 is pervasively upregulated and clinically detrimental in gastric cancer

**FIGURE 2:**
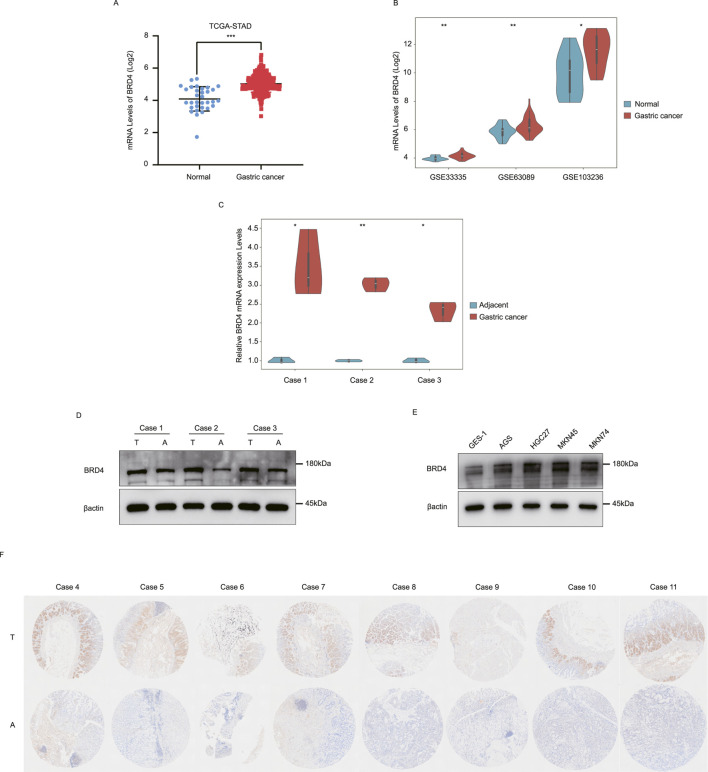
BRD4 expression is significantly higher in gastric cancer tissues and cells compared to normal gastric tissues and epithelial cells. **(A)** The TCGA-STAD dataset indicates that BRD4 expression is significantly elevated in GC tissues compared to normal gastric tissues. **(B)** Multiple GEO datasets (GSE33335, GSE63089, and GSE103236) demonstrate that BRD4 expression is significantly higher in GC tissues than in adjacent normal tissues. **(C)** RT-qPCR results reveal that BRD4 mRNA levels are significantly higher in GC tissues compared to adjacent normal tissues across three paired samples (Cases 1–3). **(D)** Western blot analysis confirms higher BRD4 protein levels in GC tissues relative to adjacent normal tissues in three paired samples (Cases 1–3). **(E)** Western blot results show that BRD4 protein levels are elevated in GC cell lines (AGS, HGC27, MKN45, MKN74) compared to the normal gastric epithelial cell line (GES-1). **(F)** Immunohistochemical (IHC) staining for BRD4 in 8 paired tumor and adjacent normal tissues (Cases 4–11) shows higher BRD4 protein levels in GC tissues compared to adjacent normal tissues. Statistical method: Mann-Whitney U test **(A,B)**; Student’s t-test **(C)**. *p < 0.05, **p < 0.01, ***p < 0.001. (Abbreviations: T, tumor tissue; A, adjacent normal tissue; GC, gastric cancer; TCGA-STAD, The Cancer Genome Atlas Stomach Adenocarcinoma; GEO, Gene Expression Omnibus).

The hypothesis that BRD4 acts as an epigenetic driving factor for tumor growth implies a direct connection between its expression quantities and malignancy, so to verify this clinical relevance, we examined transcriptomic information from The Cancer Genome Atlas (TCGA) and several Gene Expression Omnibus (GEO) datasets (GSE33335, GSE63089, and GSE103236), which indicated a substantial and stable increase in BRD4 transcripts throughout tumor samples (Figures 2A,B). These computational findings were experimentally confirmed by employing RT-qPCR, WB and immunohistochemistry (IHC) on corresponding clinical samples, showing that our outcomes displayed a great amount of BRD4 protein build - up in tumor tissues while the nearby healthy mucosa showed little to no expression (Figures 2C–F). During our pathological evaluations under the microscope, we were particularly struck by the intense, almost exclusively nuclear punctate staining pattern of BRD4 in the tumor cores, which sharply contrasted with the pale, unstained background of the adjacent normal tissues. Moreover, a single - cell deconvolution analysis of dataset GSE134520 pinpointed this BRD4 excessive expression exactly in the malignant epithelial cell group leaving the surrounding immune and stromal areas untouched, which suggested a positive therapeutic opportunity for BRD4 targeted degradation approaches (Supplementary Figure S2B). This cell-type specificity is highly encouraging, as it implies that systemic targeting might spare the vital tumor-infiltrating lymphocytes required for anti-tumor immunity.

**FIGURE 3:**
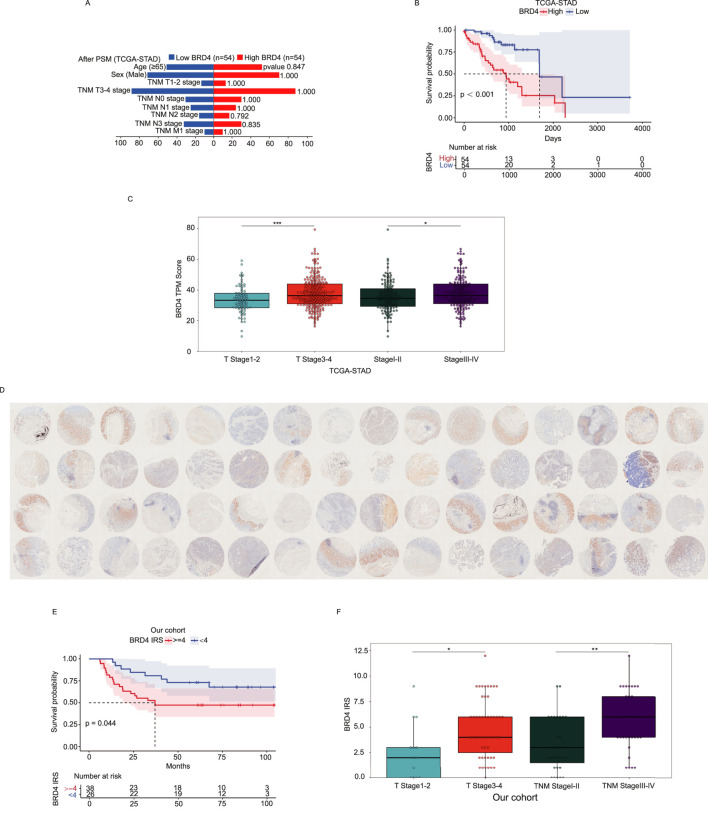
Higher BRD4 expression in gastric cancer correlates with increased malignancy, more advanced TNM stage, and poorer patient prognosis. **(A)** After Propensity Score Matching (PSM), there are no significant differences in clinicopathological features between BRD4-high and BRD4-low GC patients (TCGA-STAD cohort); **(B)** After PSM, higher BRD4 expression is associated with poorer overall survival (TCGA-STAD cohort); **(C)** BRD4 expression levels increase in patients with more advanced TNM stages (TCGA-STAD cohort); **(D)** Representative images of immunohistochemical (IHC) staining for BRD4 in 64 GC tissue samples from our independent cohort; **(E)** Kaplan-Meier survival analysis of 64 GC patients in our cohort demonstrates that higher BRD4 expression correlates with a worse prognosis (IRS ≥4 vs. < 4); **(F)** BRD4 protein expression levels are higher in patients with more advanced TNM stages (Our cohort). Statistical method: Chi-square test **(A)**; Log-rank test **(B,E)**; Mann-Whitney U test **(C,F)**. *p < 0.05, **p < 0.01, ***p < 0.001. (Abbreviations: GC, gastric cancer; PSM, propensity score matching; TNM, tumor-node-metastasis; IHC, immunohistochemistry; IRS, immunoreactive score).

The conversion of these molecular discoveries into clinical results demonstrated a strong connection between BRD4 expression and patients’ prognoses; initial studies on survival showed that patients having high BRD4 expression tended to have worse outcomes and this result achieved statistical importance (p < 0.001) after Propensity Score Matching was utilized to regulate for interfering elements like age and initial TNM stage (Figures 3A,B; Supplementary Figures S2B,C). Furthermore, it was found that high BRD4 expression served as an individualized prognostic element for severe diseases and had substantial correlations with deeper muscle layer invasion, more progressed TNM stages, and bad histological distinction (Figure 3C; Supplementary Figure S2F). Our clinical group confirmed these findings as patients with high BRD4 protein expression (IRS≥4) had greatly decreased survival and more advanced TNM stages (Figures 3D–F); moreover, BRD4 expression was considerably increased in male patients (Supplementary Figure S2D) and in those with *Helicobacter* pylori-negative gastric cancer (Supplementary Figure S2E).

### Disrupting the BRD4-MYC-BCL2 axis induces profound tumor cell collapse

**FIGURE 4:**
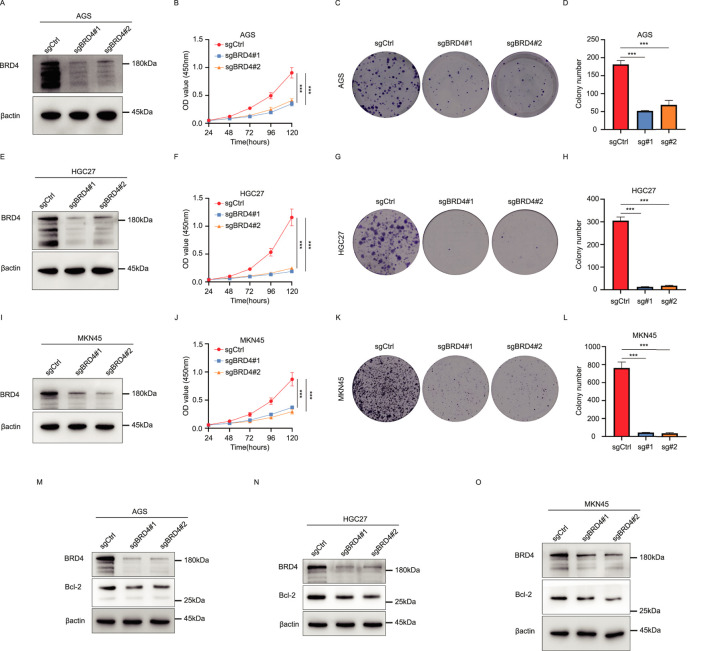
Genetically removing BRD4 substantially weakens the proliferative ability of gastric cancer cells and at the same time upsets the MYC-BCL2 anti-apoptotic signaling pathway. **(A,E,I)** WB assay confirm the CRISPR-Cas9-mediated knockout of BRD4 (sgBRD4) in the AGS **(A)**, HGC27 **(E)**, and MKN45 **(I)** cell lines. **(B,F,J)** The Cell Counting Kit-8 (CCK-8) assays indicated that after BRD4 was depleted, the proliferative capacity in AGS **(B)**, HGC27 **(F)**, and MKN45 **(J)** cells decreased significantly. **(C,D,G,H,K,L)** There are representative images and quantitative analysis of clonogenic survival assays which demonstrated that there was a substantial reduction in colony formation in AGS **(C,D)**, HGC27 **(G,H)**, and MKN45 **(K,L)** cells with BRD4 knocked out. **(M–O)** Western blot analysis showed that the level of BCL2 protein decreased considerably following BRD4 knockout in AGS **(M)**, HGC27 **(N)**, and MKN45 **(O)** cells. Statistical method: Two-way ANOVA test **(B,F,J)**; Student’s t-test **(D,H,L)**. *p < 0.05, **p < 0.01, ***p < 0.001.

Given the pervasive upregulation and poor clinical outcomes associated with BRD4, we next sought to establish a functional dependency. To clarify how gastric tumors depend on transcription driven by BRD4, we utilized CRISPR-based knockout technology in several gastric cancer cell lines like AGS, HGC27, and MKN45. The resulting changes in appearance were substantial and quick, showing that cells without BRD4 had much weaker growth ability and could barely form long lasting colonies (Figures 4A–L). Daily visual inspections of the culture flasks revealed that these BRD4-depleted cells not only ceased dividing but also exhibited pronounced morphological signs of distress, such as rounding up and diminished substrate adherence, long before colony formation was officially quantified.

Intrigued by these dramatic phenotypic changes, we aimed to figure out what was mediating the cellular collapse we noticed. To this end, we did a comparative study of the predicted MYC downstream targets (ASS1, MTDH, BCL2, IGF2BP1, ST3GAL3, TOP1MT, and NOL7) and the genes that were significantly downregulated after BRD4 broke down, which led us to spot BCL2, a well - known anti - apoptotic protein, as the main possibility (Supplementary Figure S3A–C). Subsequent Western blot analysis showed that getting rid of BRD4 genetically made the levels of BCL2 protein in gastric cancer cells drop a great deal (Figures 4M–O), so these results proved that inhibiting BRD4 pushed cells to undergo apoptosis through the BRD4 - MYC - BCL2 signaling pathway and demonstrated that BRD4 was involved not just in promoting cell growth but also in giving anti - apoptotic protection to tumor cells. Because BCL2 acts as the ultimate gatekeeper for mitochondrial outer membrane permeabilization, its collapse provides a satisfying mechanistic bridge linking epigenetic silencing directly to executioner caspase activation.

### Targeted degradation via ARV771 yields potent, safe anti-tumor efficacy

**FIGURE 5:**
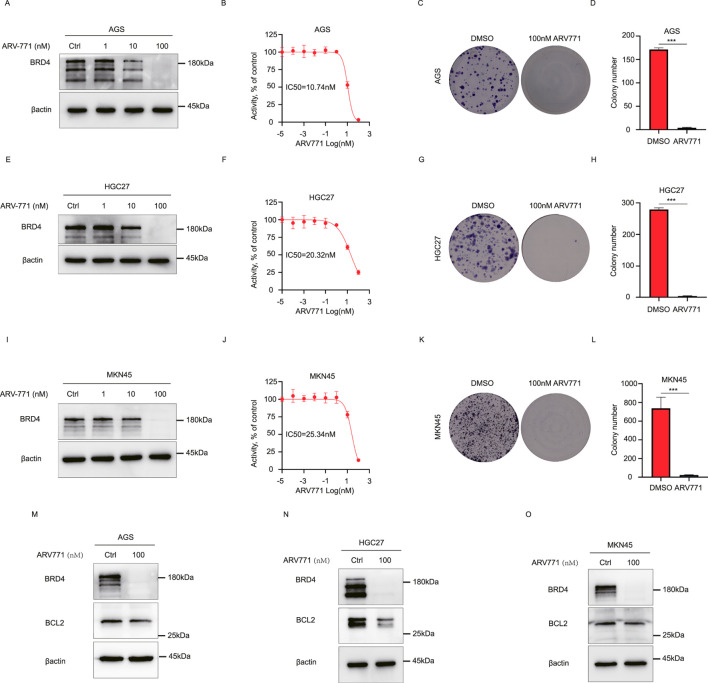
The PROTAC degrader ARV771 exerts potent anti-tumor effects by eliminating BRD4 and reducing BCL2 protein expression *in vitro*. **(A,E,I)** Western blot analysis showed that there was a dose-dependent degradation of BRD4 in AGS **(A)**, HGC27 **(E)**, and MKN45 **(I)** cells after being treated with ARV771; **(B,F,J)** Dose-response curves indicated that ARV771 had inhibitory effects on cell viability and the half maximal inhibitory concentrations (IC50) were calculated to be in the nanomolar range for AGS **(B)**, HGC27 **(F)**, and MKN45 **(J)** cell lines; **(C,D,G,H,K,L)** Clonogenic survival assays demonstrated that treating with 100 nM ARV771 led to a nearly complete loss of colony forming potential in AGS **(C,D)**, HGC27 **(G,H)**, and MKN45 **(K,L)** cells; **(M–O)** Immunoblot analysis confirmed that the degradation of BRD4 mediated by ARV771 corresponded to a simultaneous decrease in the expression of the anti-apoptotic protein BCL2 in AGS **(M)**, HGC27 **(N)**, and MKN45 **(O)** cells. Statistical method: Non-linear regression analysis **(B,F,J)**; Student’s t-test **(D,H,L)**. *p < 0.05, **p < 0.01, ***p < 0.001. (Abbreviations: PROTAC, proteolysis targeting chimera).

With the functional dependency established, we explored pharmacological interventions. In March 2025, the positive outcomes from the Phase III clinical trial regarding the PROTAC known as ARV471 were disclosed, which showed that when combined with fulvestrant it considerably enhanced progression free survival (PFS) thus having great clinical significance (Ma and Zhou, 2025). Due to the critical function of BRD4 in promoting proliferation, we created ARV771, a PROTAC molecule designed to trigger the ubiquitination and ensuing proteasomal degradation of BRD4. *In vitro*, it demonstrated strong activity reaching nearly complete protein breakdown at a concentration of 100 nM and exhibiting cytotoxicity with IC50 values within the single - digit nanomolar range across all tested cell lines (Figures 5A–L). This remarkable efficacy underscores the unique “event-driven” pharmacology of degraders, which act catalytically to repeatedly eliminate targets, unlike traditional “occupancy-driven” small molecule inhibitors that require sustained high concentrations to function. In line with our mechanistic assumption, the treatment with ARV771 effectively repressed BCL2 expression (Figures 5M–O).

**FIGURE 6:**
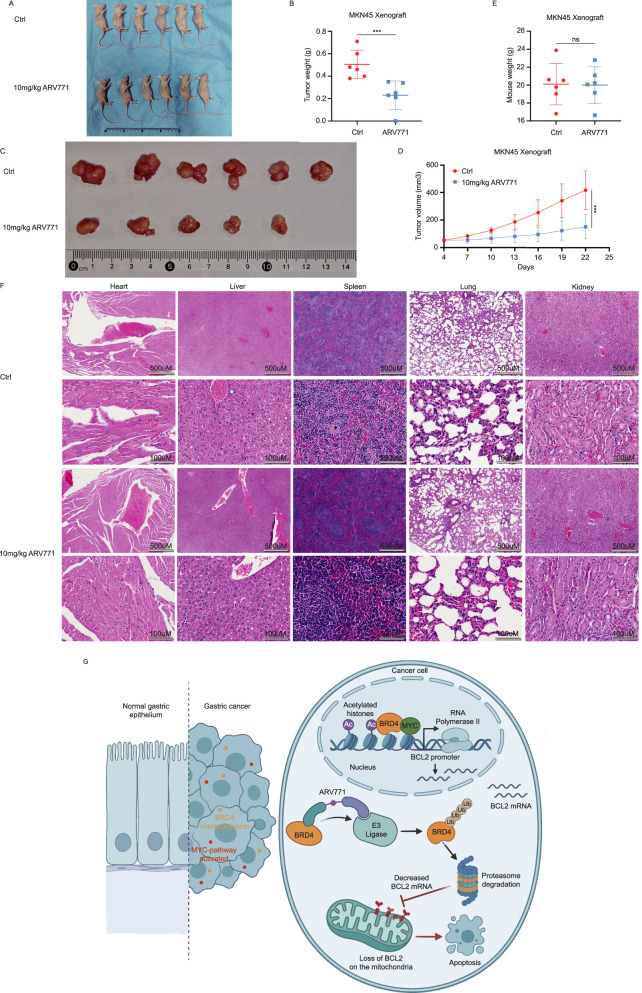
Systemic administration of ARV771 effectively inhibits tumor progression in a gastric cancer xenograft model without obvious *in vivo* toxicity. **(A,B)** *In vivo* analysis showed that there was a substantial decrease in terminal tumor sizes **(A)** and masses **(B)** after treating with ARV771, macroscopic pictures of the removed subcutaneous xenografts **(C)** and the matching tumor growth graphs **(D)** indicated that the tumors had obviously stopped growing in the group treated with ARV771 when compared to the vehicle control group. **(E)** The terminal mice’s body weight suggested that there wasn't any significant systemic toxicity during the whole treatment time. **(F)** The typical Hematoxylin and Eosin (H&E)-stained slices of main visceral organs (heart, liver, spleen, lung, kidney) verified that the physiology could tolerate ARV771 and there was no structural harm related to its administration. **(G)** A general view of the study process was given in the form of a diagram. Statistical method: Student's t-test **(B,E)**; Two-way ANOVA test **(D)**. *p < 0.05, **p < 0.01, ***p < 0.001, ns, not significant. (Abbreviations: H&E, hematoxylin and eosin).

To definitively confirm the therapeutic potential of this strategy, finally, an *in vivo* evaluation using a mouse xenograft model showed that when ARV771 was administered intravenously, it brought about a substantial hold up in the growth of tumor volume and mass compared to the vehicle treated controls (Figures 6A–D). Moreover, during the whole experiment, the body weights of the animals given ARV771 remained the same as those of the control group (Figure 6E) and crucially, such a strong anti - tumor result was seen without any noticeable histopathological issues in the heart, liver, spleen, lungs, and kidneys (Figure 6F). Beyond the macroscopic organ evaluations, we also routinely noted that the treated mice maintained normal grooming behaviors, physical mobility, and feeding habits throughout the monitoring period, further reinforcing the agent’s excellent tolerability profile. This proved that the system-wide safety situation related to PROTAC led BRD4 breakdown was good. A schematic diagram depicting the study workflow is shown in Figure 6G.

## Discussion

The development of gastric cancer is a complex multi - factor process which involves environmental elements, long - term inflammation often triggered by *H. pylori* infection and gradual genetic changes (Yong et al., 2015; Noto et al., 2019), and even though well - known signaling routes like MAPK and PI3K/Akt were considered to contribute to tumor growth and spread (Ren et al., 2023; Xu et al., 2018), the MYC signaling route acts as a key transcriptional controller and by attaching to E-box patterns it adjusts a wide range of gene expression networks which govern cellular activities such as the progress of the cell cycle, metabolic adjustment (for instance, depending on glutamine) (Walz et al., 2014; Nie et al., 2012; Hsu et al., 2015; Dong et al., 2020; Li and Simon, 2013; Casey et al., 2017; Li et al., 2023).

The role of MYC in promoting cancer has been well - known for a long time, but inhibiting it pharmacologically has been a great challenge for many years mainly because there are no targetable structural pockets (Whitfield and Soucek, 2025; Duffy et al., 2021), so people had to shift their strategy to target the epigenetic mechanisms that keep its abnormal activity going, our bioinformatics analysis showed that the MYC signaling pathway was highly active in gastric tumors and this was closely related to the overexpression of its main co - activator, BRD4, an integrated analysis of multi - omics data from The Cancer Genome Atlas (TCGA) and Gene Expression Omnibus (GEO) databases confirmed that BRD4 was consistently and significantly more abundant in gastric tumor tissues than in nearby normal mucosa and this computational result was verified by experiments such as RT - qPCR, Western blotting, and immunohistochemical (IHC) analyses, also single - cell transcriptomic profiling drew out a clear spatial expression pattern showing that BRD4 was mostly expressed in malignant epithelial cells with very little expression in infiltrating immune cells and benign glandular structures, this local overexpression indicated that BRD4 gave gastric cancer cells a selective advantage making it an attractive target for treatment.

The maintenance of the malignant condition by this epigenetic reader occurs via its essential part in transcriptional regulation since being a central element of the transcription apparatus, BRD4 attaches to acetylated chromatin mainly in super - enhancer areas thus drawing RNA polymerase II to promote the effective extension of crucial oncogenic transcripts (Kanno et al., 2014; Donati et al., 2018), and this regulatory system was marked by a vital functional interdependency between BRD4 and MYC as specifically, BRD4 took up the super - enhancers controlling the MYC region causing its over - activation and creating a situation of MYC dependence in the tumor (Tan et al., 2018; Ba et al., 2017), also BRD4 formed cooperative co - activator complexes with proteins like WDR5 which greatly increased MYC’s transcriptional productivity (Thomas et al., 2019), and our Gene Ontology (GO) enrichment analysis supported these mechanistic understandings showing that increased BRD4 expression in gastric cancer was strongly related to RNA polymerase II - dependent transcription elongation complexes, so even though there were difficulties in directly targeting MYC, restraining BRD4 was a powerful and indirect treatment approach to disturb the MYC oncogenic process at its epigenetic base.

The clinical importance of molecular drivers is basically decided by how they affect patient results and in solid tumors, abnormal expression of certain regulatory proteins has always shown great prognostic worth like TACC3 in gliomas (Sun et al., 2017), peroxiredoxins in endometrial carcinomas (Byun et al., 2018), particular proteasome components (PSMD11/14) in pancreatic cancer (Yang et al., 2025), and zinc transporters in hepatocellular carcinoma (Bitirim, 2021) and based on the findings in hepatocellular carcinoma where BRD4 overexpression was a dependable sign of recurrence and decreased survival (Zhang et al., 2015) and in breast cancer where its quantity was inversely related to T - bet + tumor - infiltrating lymphocytes (TILs) and disease free survival (Lee et al., 2018), we assumed that a similar prognostic system might be present in gastric cancers.

Through our in depth examination of TCGA clinical metadata, which was supported by an independent immunohistochemical cohort consisting of 64 patients, it was shown that high BRD4 expression serves as a crucial indicator of negative clinical results and a substantial positive connection was found among BRD4 expression amounts, the degree of invasion, bad histological quality, and an advanced pathological phase, and it was particularly noteworthy that BRD4 expression was considerably greater in male patients and those who were *H. pylori* negative, implying possible biology functions related to specific causes that are worthy of further exploration, so all these findings confirmed that BRD4 is a strong predictive biomarker for separating patients into different groups, thus broadening its function far beyond just being an epigenetic controller.

To set up a cause effect connection between BRD4 and the ability to cause tumors, we examined the function of BRD4 *in vitro* through genetic ablation and found that interfering with BRD4 expression in several gastric cancer cell lines (AGS, HGC27, MKN45) led to a remarkable decrease in cell proliferation and the capacity to form colonies, showing how crucial it was for maintaining the viability of tumor cells; then, to figure out the mechanisms underlying this, we carried out transcriptomic profiling and discovered that after BRD4 was depleted, there was a significant reduction in the MYC signaling axis among the possible MYC target genes like ASS1, MTDH, and BCL2, and BCL2, which regulates anti - apoptosis, had the largest drop in expression since BCL2 plays a vital part in preventing apoptosis, so these results pointed out a crucial BRD4-MYC-BCL2 signaling axis, and this mechanism from epigenetics to apoptosis seems to be a preserved cancer - promoting pathway because similar findings were reported in salivary gland adenoid cystic carcinoma (Wang et al., 2017) and cutaneous squamous cell carcinoma (Xiang et al., 2018), also MYC is a main controller of cell cycle progress and metabolic adjustment (Sabò et al., 2014), and its coordinated increase in BCL2 offers a necessary anti - apoptotic protection enabling fast, unrestricted growth (Mcmahon, 2014), furthermore, the clinical importance of this axis is emphasized by the bad prognosis related to MYC/BCL2 co-expression known as double hit biology in diffuse large B-cell lymphoma (DLBCL) (Johnson et al., 2012; Hu et al., 2013; Pedersen et al., 2012), and altogether, these data showed that disrupting the BRD4 dependent BCL2 upregulation mechanism through therapy efficiently blocked a major survival path in tumor cells.

After establishing the biological and clinical justifications, the translation of these understandings requires the creation of ideal pharmacological tools and even though traditional BET inhibitors like JQ1, OTX015, and AZD5153 had shown strong effectiveness by competing for the BRD4 binding pockets and causing cell cycle stoppage or ferroptosis in melanoma and neuroendocrine prostate cancer models (Li et al., 2025a; Li et al., 2025b; Miller et al., 2021; Zhang et al., 2025), their use in clinics was often limited due to insufficient pathway suppression and toxicity that restricted dosages so the appearance of PROTAC (Proteolysis-Targeting Chimera) technology was a game - changing method as this approach made it possible to recruit E3 ubiquitin ligases to bring about the targeted breakdown of the protein instead of simply blocking its active site (He et al., 2020; Li and Song, 2020) and the clinical progress of oral PROTACs such as ARV110 and ARV471 had already brought about revolutionary results in hormone-driven cancers with recent Phase III trial data for ARV471 showing substantial enhancements in progression free survival (Ma and Zhou, 2025; Xi et al., 2022; Zou et al., 2024) and in the same way, targeted BRD4 degraders like ARV825 were found to quickly cause G1 cell cycle arrest in cholangiocarcinoma (Lu et al., 2019).

By making use of this event-driven pharmacological method, we looked into the BRD4-targeting degrader ARV771 (Deng et al., 2022), which had not been well - studied before in gastric cancer; our in - vitro research showed that ARV771 brought about considerable cytotoxicity and when we treated cells with ARV771 at low - nanomolar concentrations (100 nM), it led to almost complete ubiquitination and proteasomal breakdown of BRD4 in the AGS, HGC27, and MKN45 cell lines; this breakdown of BRD4 was in line with what was expected in terms of molecular outcomes such as a large decrease in the MYC - dependent anti - apoptotic factor BCL2; to evaluate its therapeutic potential in living organisms, we administered ARV771 systemically through tail - vein injection and it worked very effectively, significantly slowing the growth of stomach tumors and greatly reducing the final tumor load; histopathological examination of major organs (heart, liver, spleen, lungs, and kidneys) indicated an excellent systemic safety situation, showing that there were no obvious signs of damage or toxicity.

## Conclusion

This study provides evidence that BRD4 is a crucial epigenetic factor controlling the progress of gastric cancer as it spurred tumor growth and also having higher levels of BRD4 indicated that the disease would be aggressive and the clinical results would be poor; BRD4 contributed to the development of tumors by keeping the MYC - BCL2 survival mechanism stable; the PROTAC degrader named ARV771 worked well in disrupting these cancer-causing processes leading to substantial tumor shrinkage in both *in vitro* and *in vivo* models; so using drugs to break down BRD4 offered a hopeful multiple approach treatment plan which could make the existing targeted therapy regimens for gastric cancer more effective.

### Limitations

Our results offered strong proof that BRD4 could be targeted for therapy, but the study had certain inborn flaws as although the effectiveness of ARV771 could be shown in controlled *in vitro* situations and in immune-deficient xenograft models, translating it into the clinic remained a great difficulty and the long-term whole-body impacts and possible compensating resistance mechanisms caused by prolonged PROTAC exposure required thorough long-term research; moreover, even though we had clarified the main BRD4-MYC-BCL2 signaling pathways, the complex and changing interactions between BRD4 and various stromal and immune cell types in the complete human tumor microenvironment needed further exploration with high resolution and spatial transcriptomic methods and finally considering the diversity of treatment responses, finding solid and predictive biomarkers to group patients who were most likely to gain benefits from BRD4 degradation was a crucial condition for successfully implementing this strategy in the clinic.

## Data Availability

The original contributions presented in the study are included in the article/Supplementary Material, further inquiries can be directed to the corresponding authors.

## Supplementary material

SUPPLEMENTARY FIGURE S1 The BRD4 and MYC signaling pathways are hyperactivated, and MYC pathway activation is more pronounced in gastric cancer tissues with higher BRD4 levels. **(A)** Gene Set Enrichment Analysis (GSEA) showing the top five significantly upregulated HALLMARK pathways in gastric cancer (GC) tissues compared to adjacent normal tissues in the TCGA-STAD dataset; **(B)** GSEA demonstrating that our custom-defined BRD4 pathway is significantly upregulated in GC tissues compared to normal tissues in the TCGA-STAD dataset; **(C)** The top ten significantly upregulated KEGG pathways in BRD4-high GC tissues from the GSE19826 dataset; **(D)** The top ten significantly upregulated Gene Ontology Cellular Component (GO-CC) terms in BRD4-high GC tissues from the GSE51575 dataset; **(E)** GSEA showing that the MYC pathway is significantly activated in BRD4-high GC tissues compared to BRD4-low tissues in the GSE19826 dataset. Statistical method: Hypergeometric test **(C,D)**; Empirical permutation test **(A,B,E)**. (Abbreviations: T, tumor tissue; A, adjacent normal tissue.).

SUPPLEMENTARY FIGURE S2 BRD4 is highly expressed in gastric cancer cells, and its expression level correlates with multiple clinicopathological features. **(A)** Single-cell RNA sequencing data (GSE134520) shows higher BRD4 expression levels in malignant gastric epithelial cells; **(B)** Comparison of clinicopathological features between BRD4-high and BRD4-low GC patients before Propensity Score Matching (PSM) in the TCGA-STAD cohort; **(C)** Kaplan-Meier overall survival curves comparing BRD4-high and BRD4-low GC patients before PSM in the TCGA-STAD cohort; **(D)** The GSE13195 dataset shows higher BRD4 expression levels in male GC patients; **(E)** The GSE13195 dataset demonstrates higher BRD4 expression levels in H. pylori-negative GC patients; **(F)** The GSE103236 dataset reveals that BRD4 expression levels are significantly higher in more poorly differentiated GC tissues. Statistical method: Chi-square test **(B)**; Log-rank test **(C)**; Mann-Whitney U test **(D,E,F)**. *p < 0.05, **p < 0.01, ***p < 0.001.

SUPPLEMENTARY FIGURE S3 The anti-apoptotic gene BCL2 is a downstream target co-regulated by BRD4 and MYC. **(A)**Consensus prediction from multiple chromatin binding databases identifying downstream MYC target genes, including ASS1, MTDH, BCL2, IGF2BP1, ST3GAL3, TOP1MT, and NOL7; **(B)** Transcriptomic profiling from the GSE119241 dataset showing 48 genes consistently downregulated in RIVA and U2932 cells following BRD4 degradation; **(C)** Overlap analysis between the 7 predicted MYC downstream targets and the 48 genes downregulated upon BRD4 degradation identifies the anti-apoptotic gene BCL2 as a crucial convergent target.
