# Supplementary material for: Targeting BRD4 in gastric cancer: promoting apoptosis and suppressing tumor progression
Source: Front Pharmacol. 2026 Jun 24;17:1835830. doi: 10.3389/fphar.2026.1835830 (PMC13341472; doi:10.3389/fphar.2026.1835830)

Figure2.D

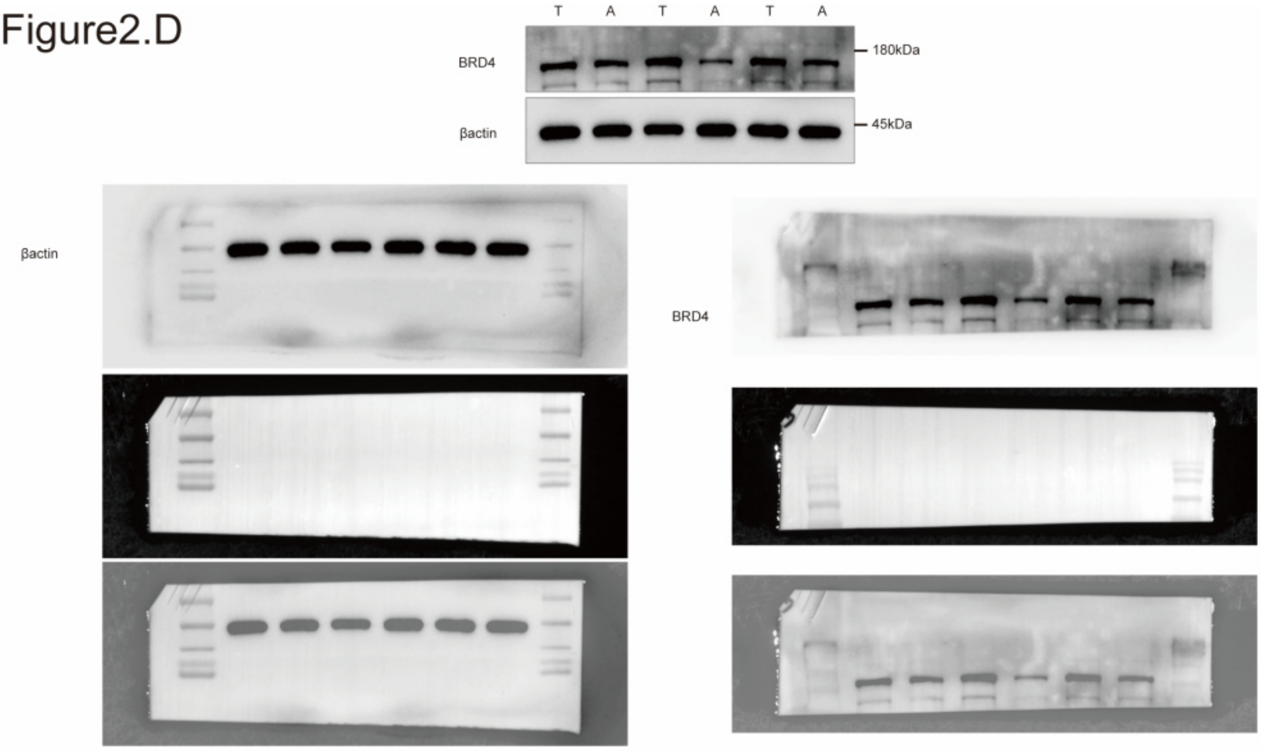

Second Repetition

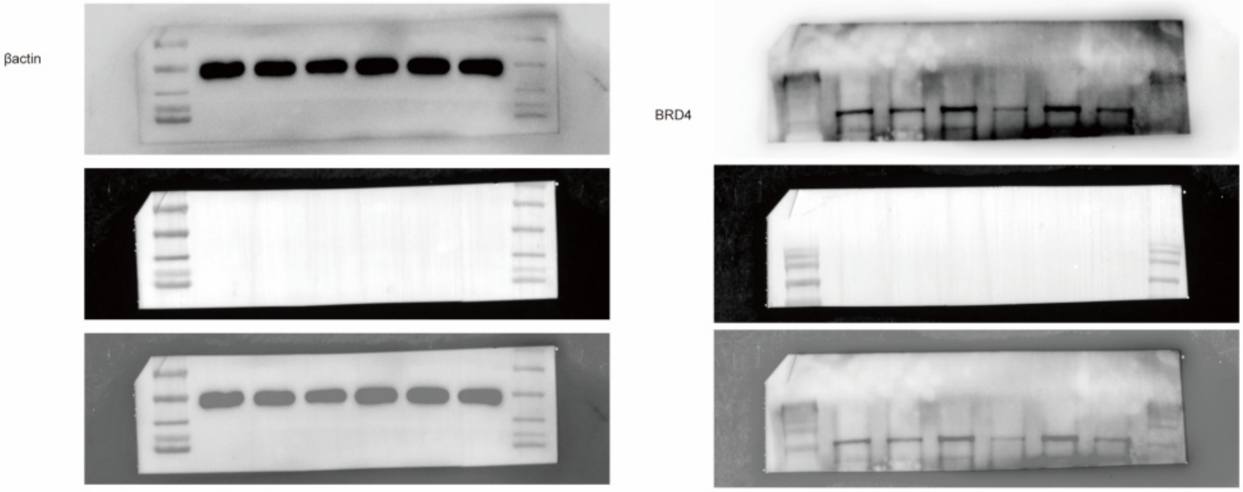

Third Repetition

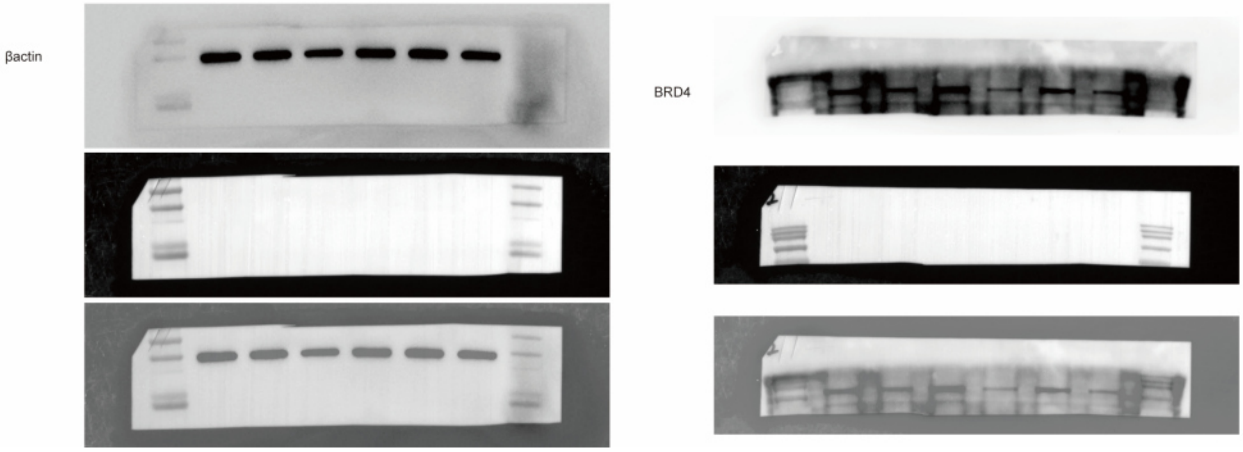

Figure2.E

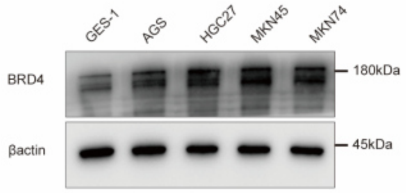

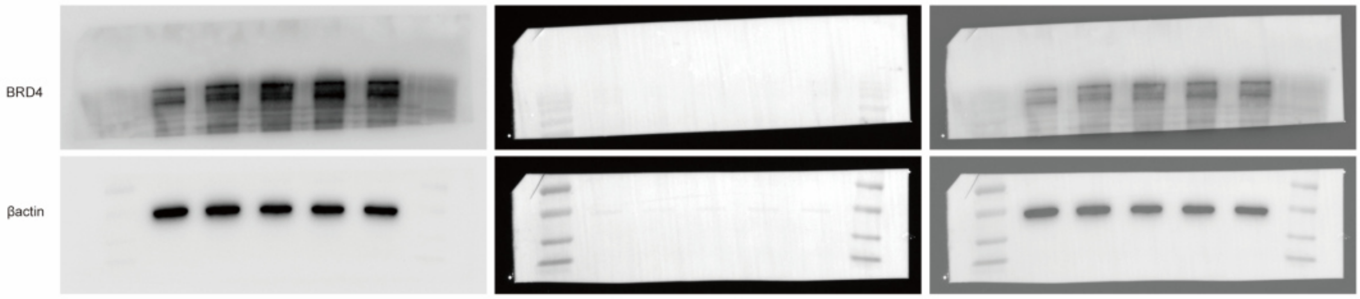

Second Repetition

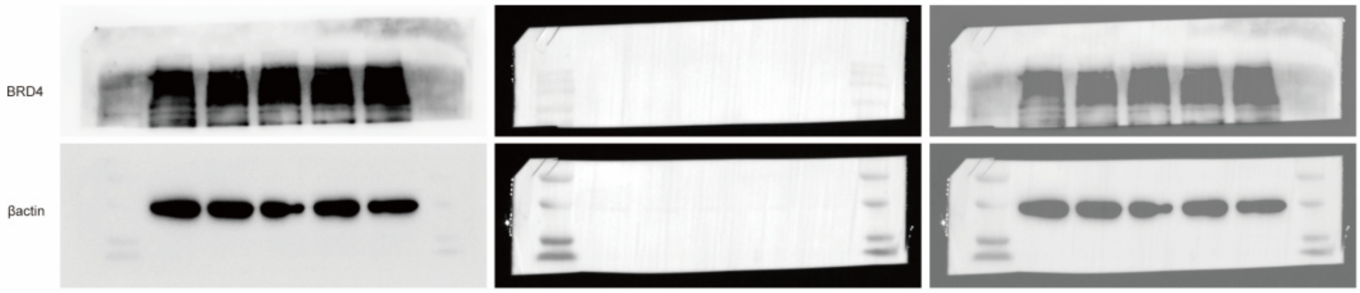

Third Repetition

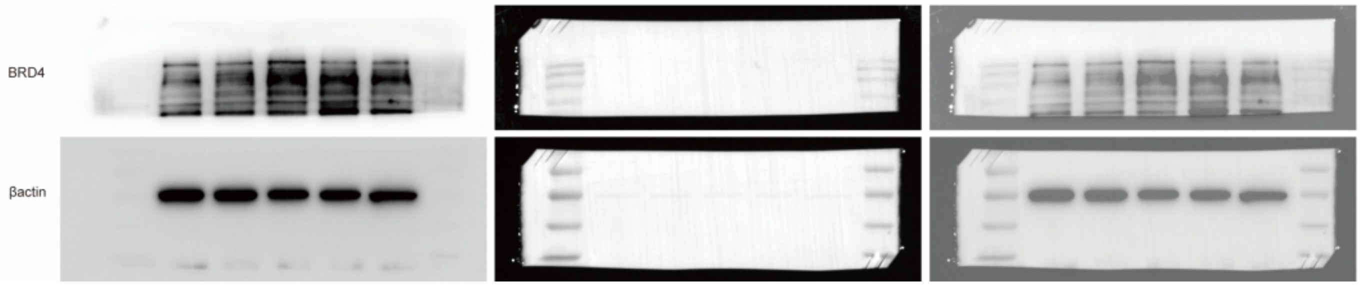

Figure3.D(First quarter)

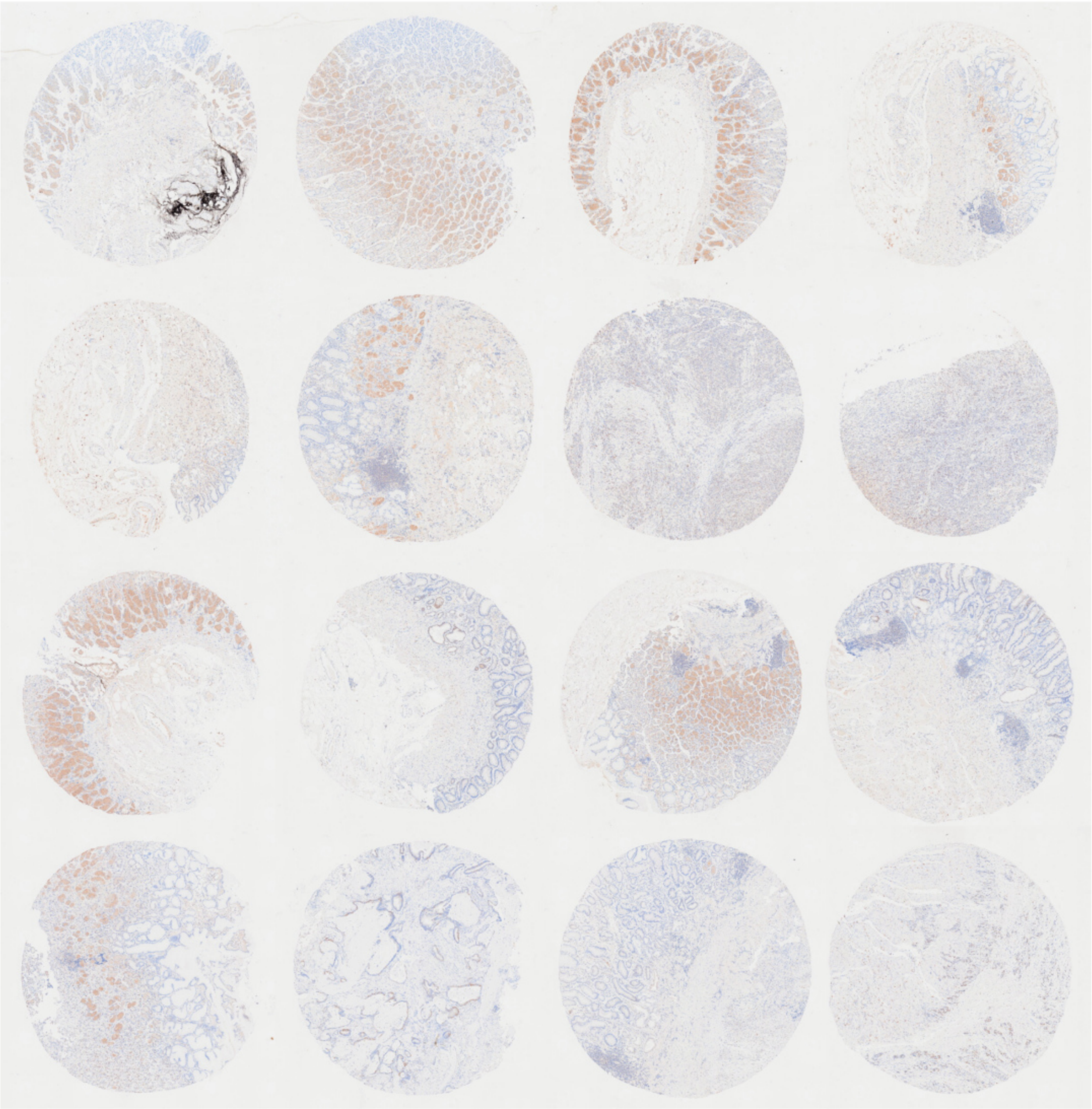

Figure3.D(Second quarter)

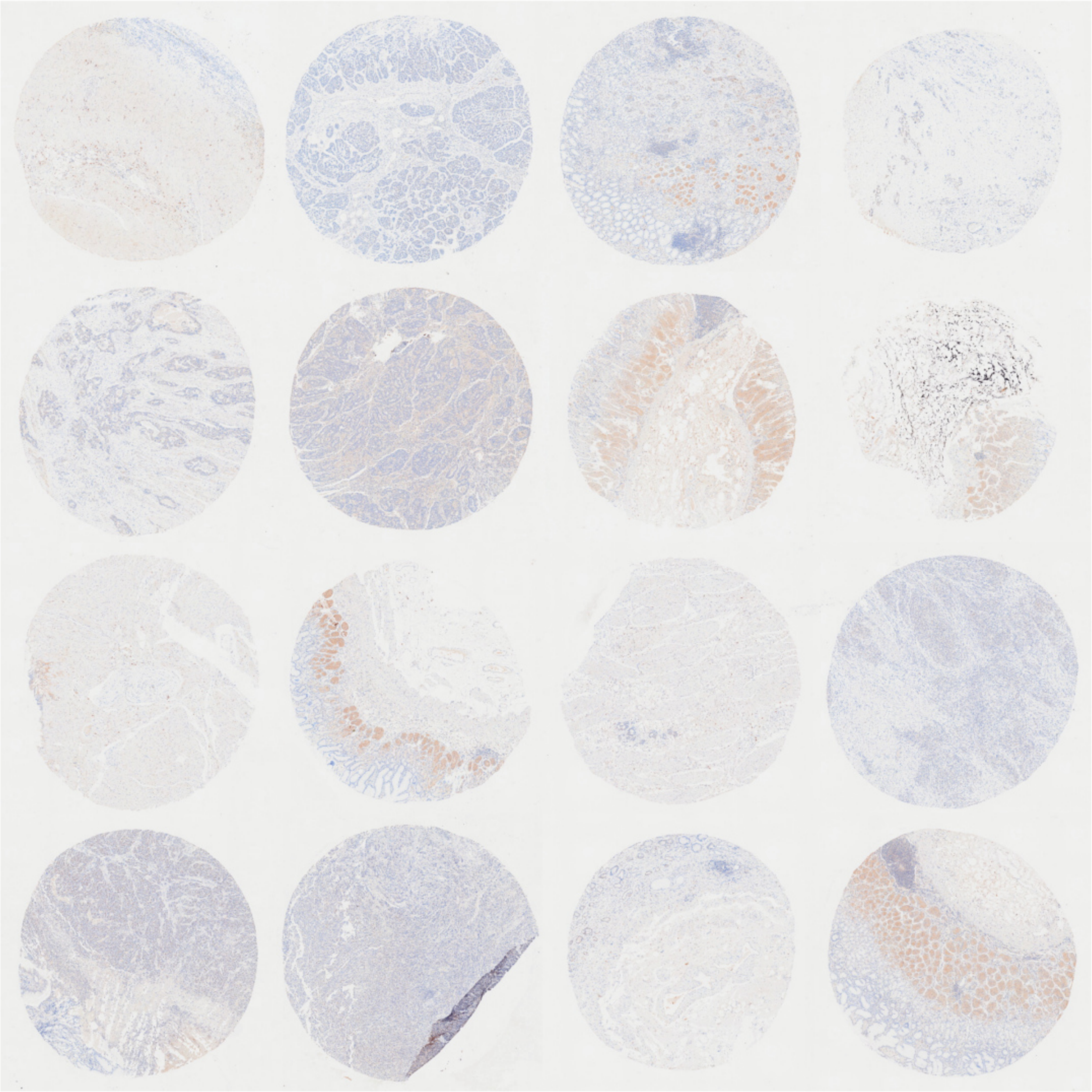

Figure3.D(Third quarter)

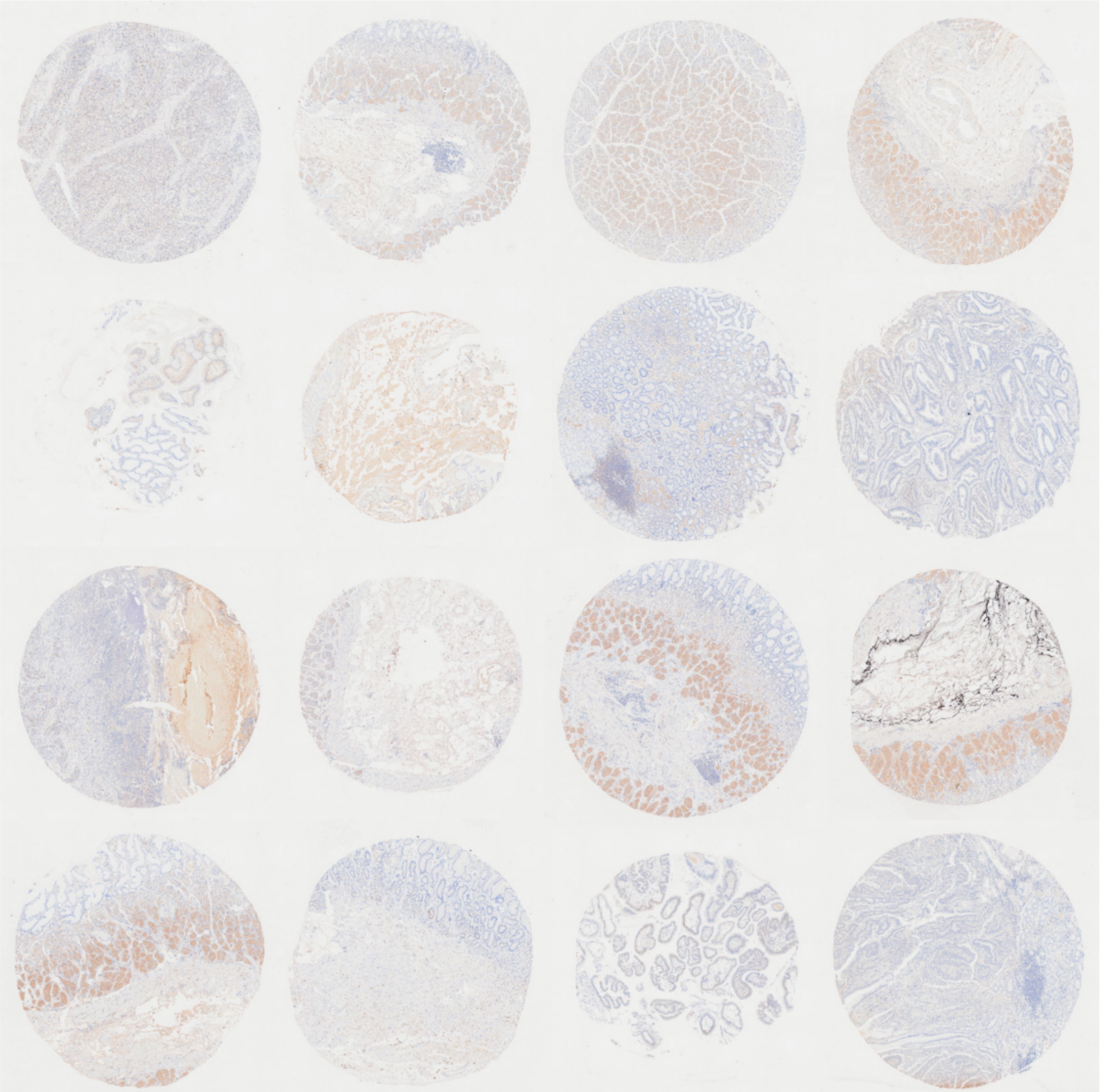

Figure3.D(Fourth quarter)

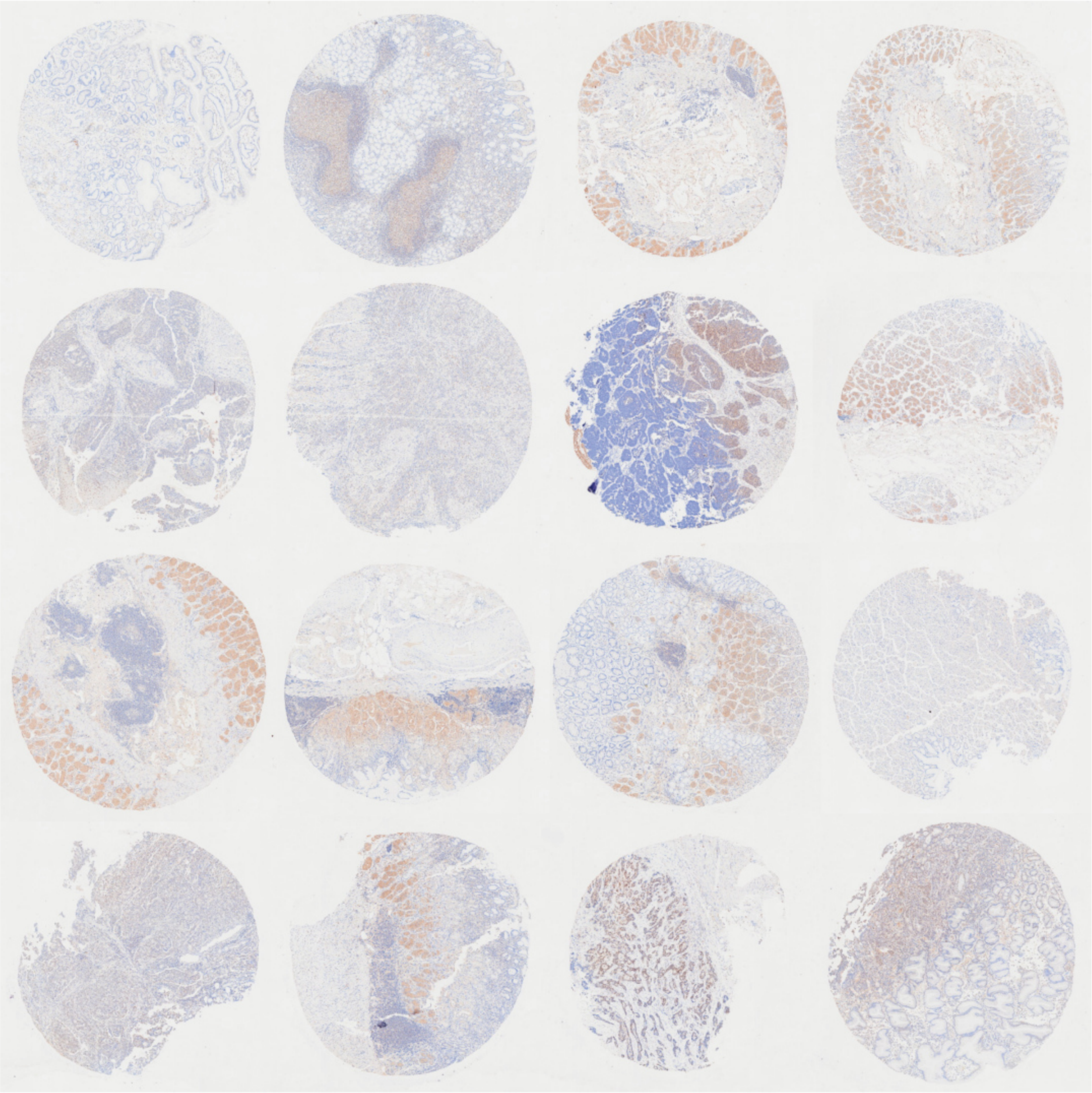

Figure4.A

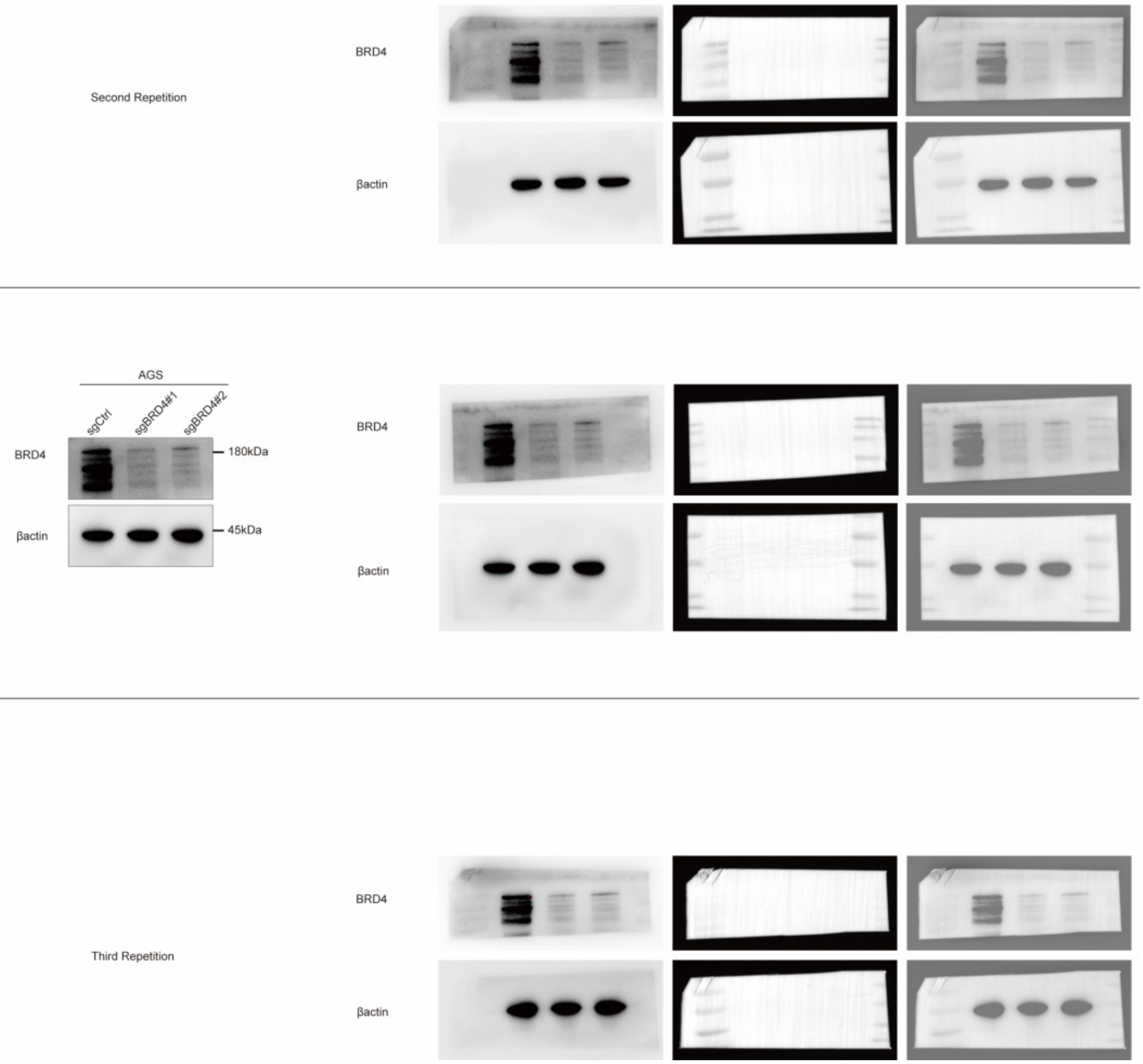

Second Repetition
BRD4
βactin
AGS
sgCtrl
sgBRD4#1
sgBRD4#2
BRD4
180kDa
βactin
45kDa
BRD4
βactin
Third Repetition
BRD4
βactin

Figure4.C

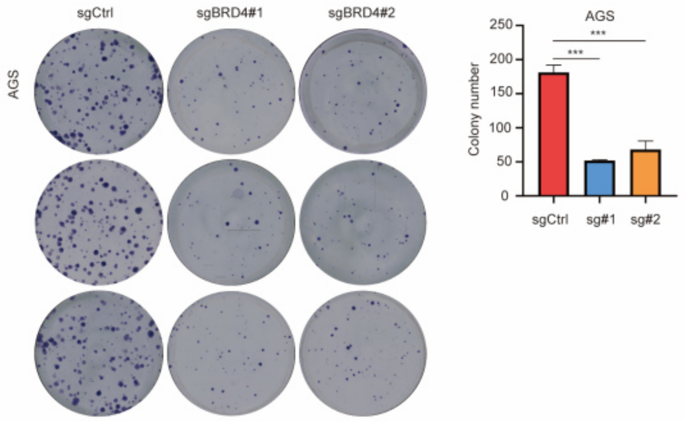

Figure6.C

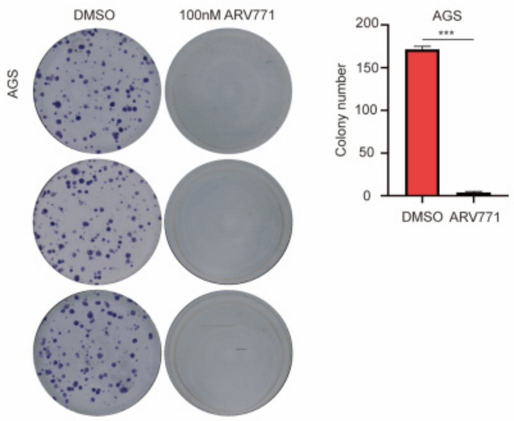

Figure4.G

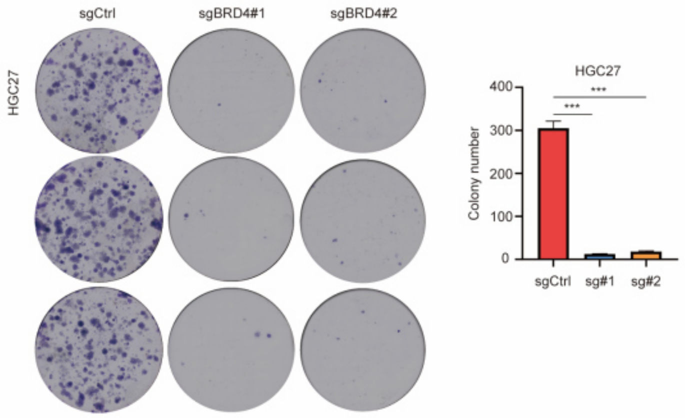

Figure6.G

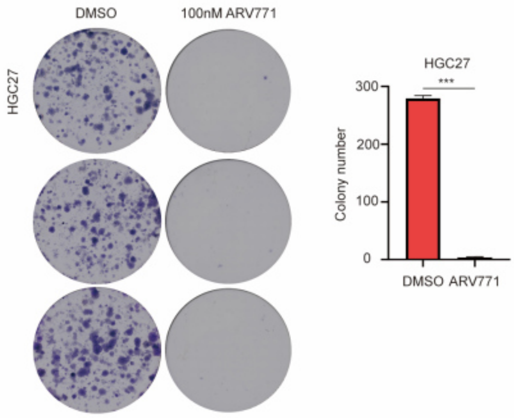

Figure4.K

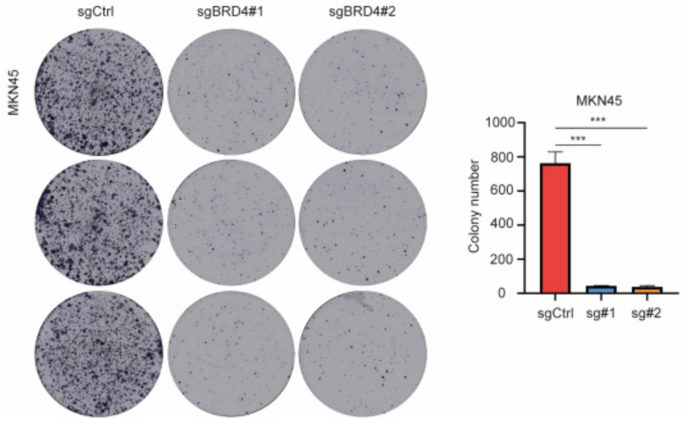

Figure6.K

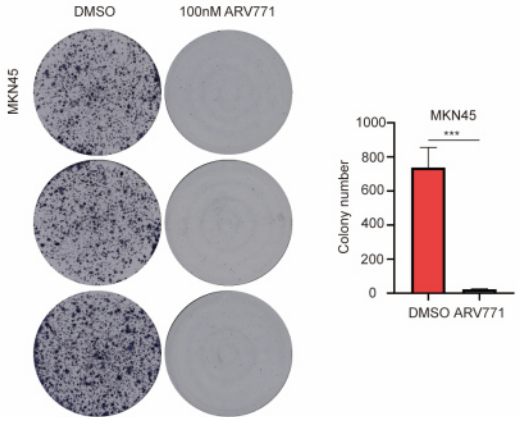

Figure4.E

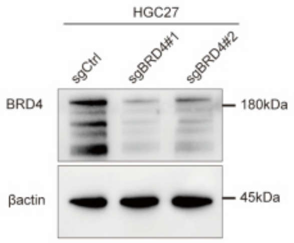
HGC27
sgCtrl
sgBRD4#1
sgBRD4#2
BRD4
180kDa
βactin
45kDa

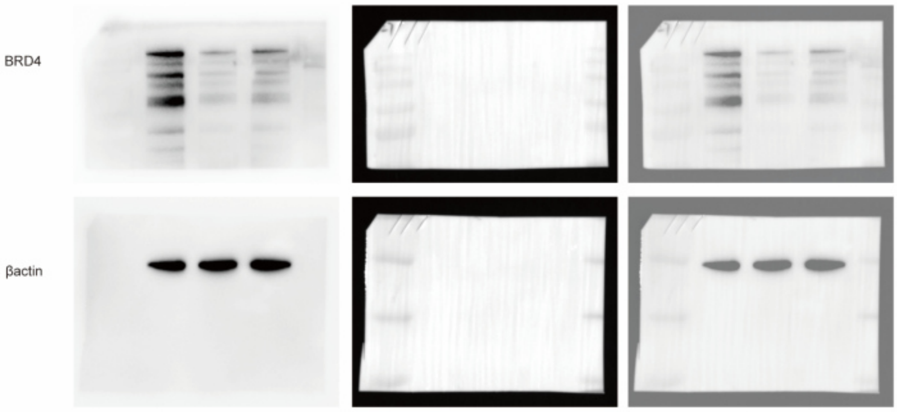
BRD4
βactin

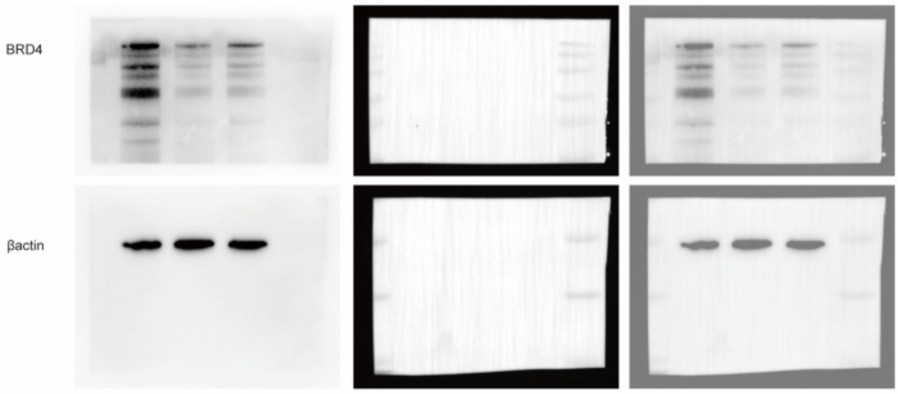
BRD4
βactin

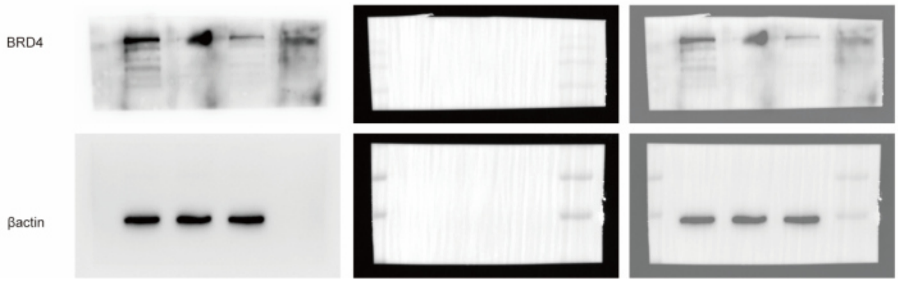
BRD4
βactin

Figure4.I

Second Repetition

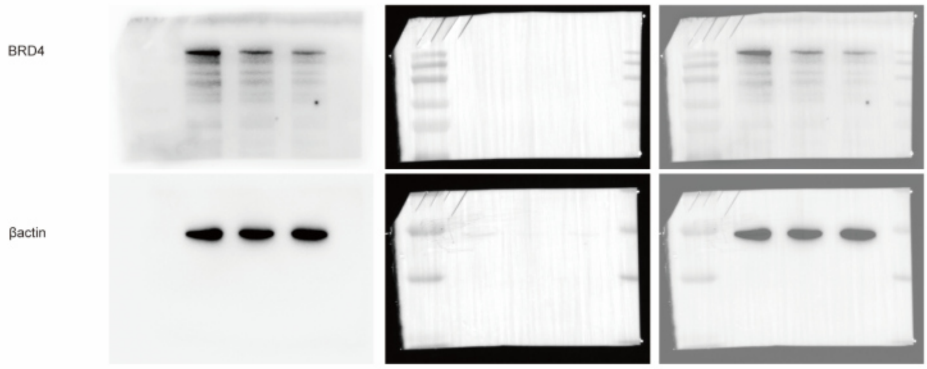

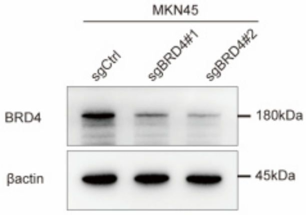

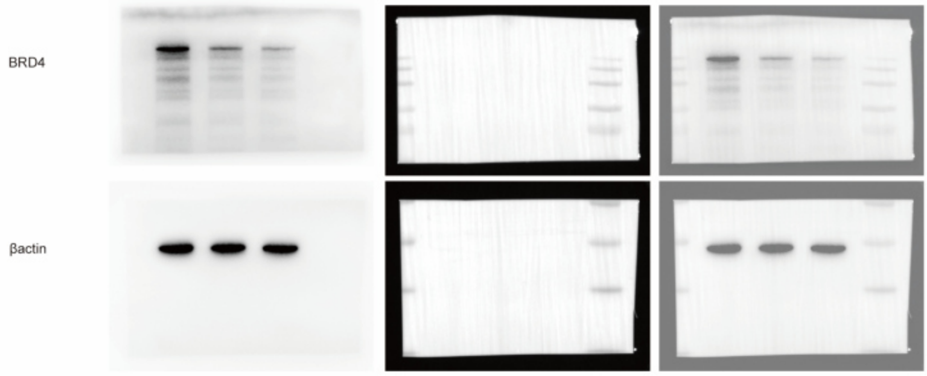

Third Repetition

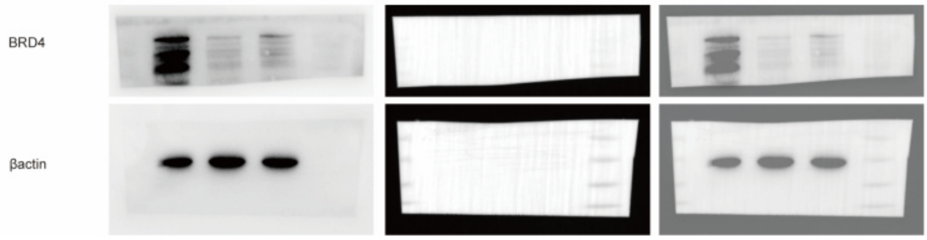

Figure4.M

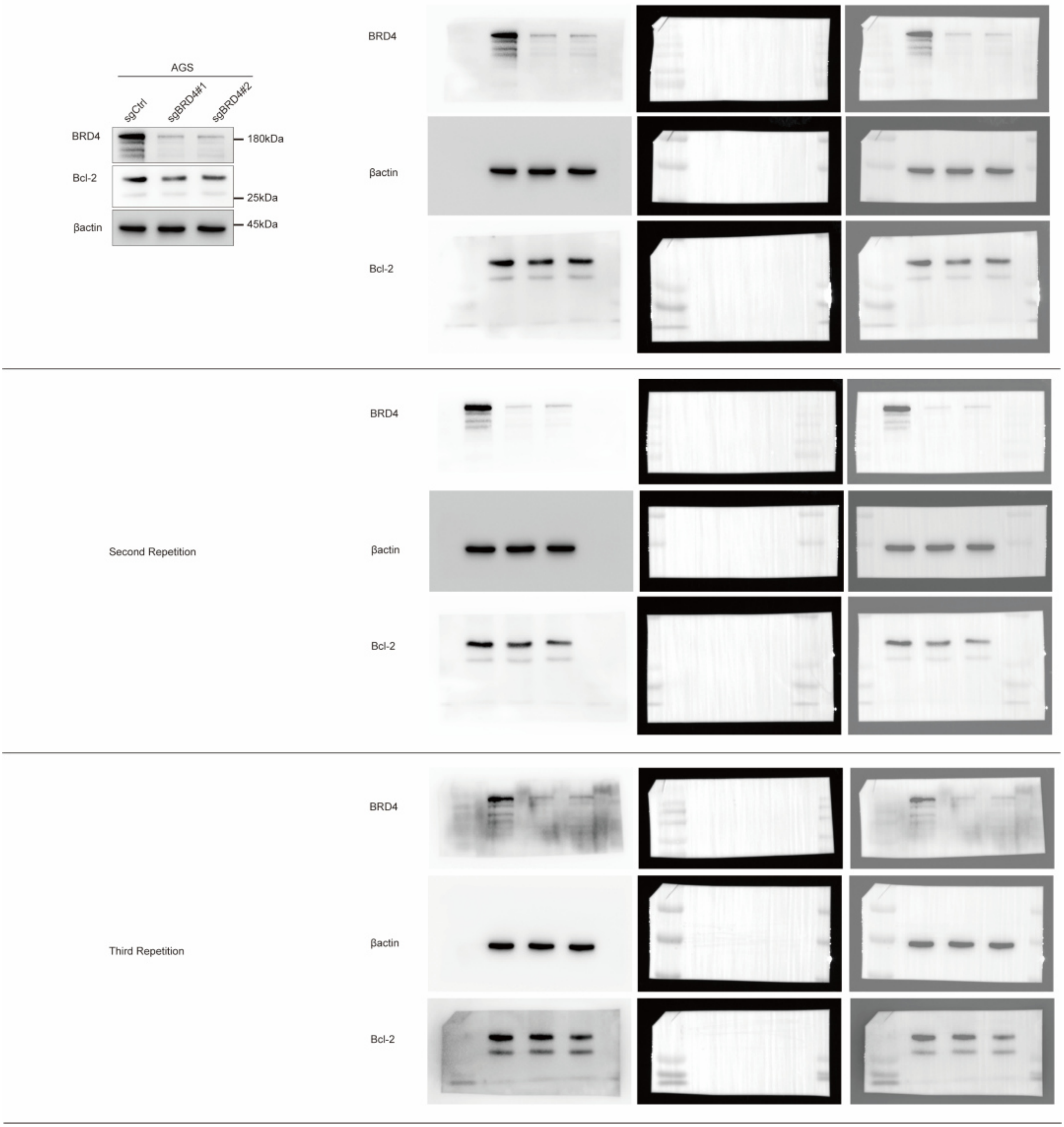
AGS
sgCtrl
sgBRD4#1
sgBRD4#2
BRD4
180kDa
Bcl-2
25kDa
βactin
45kDa
BRD4
βactin
Bcl-2
Second Repetition
BRD4
βactin
Bcl-2
Third Repetition
BRD4
βactin
Bcl-2

Figure4.N

Second Repetition

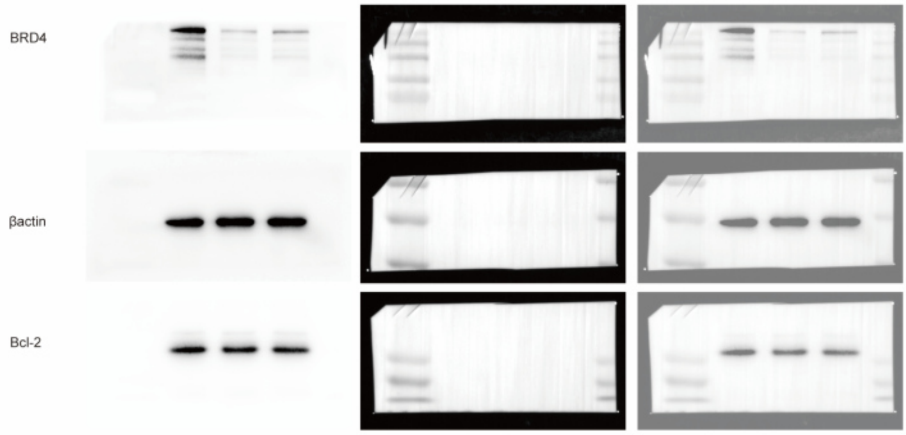

Third Repetition

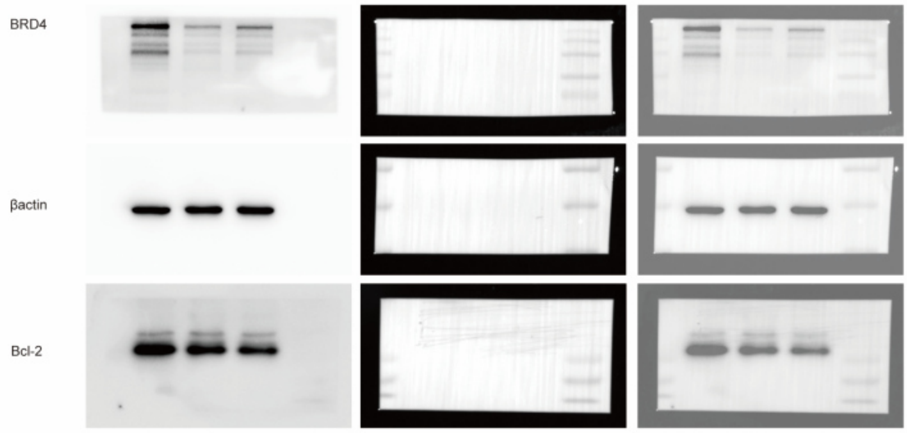

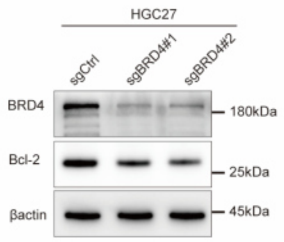

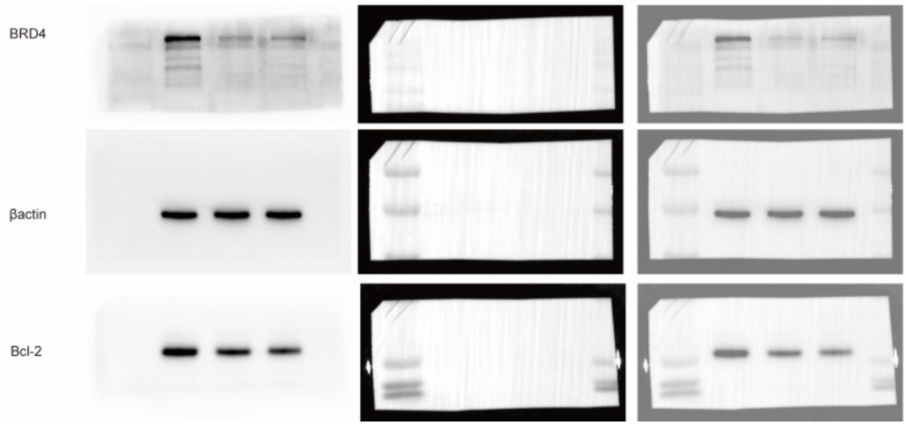

Figure4.O

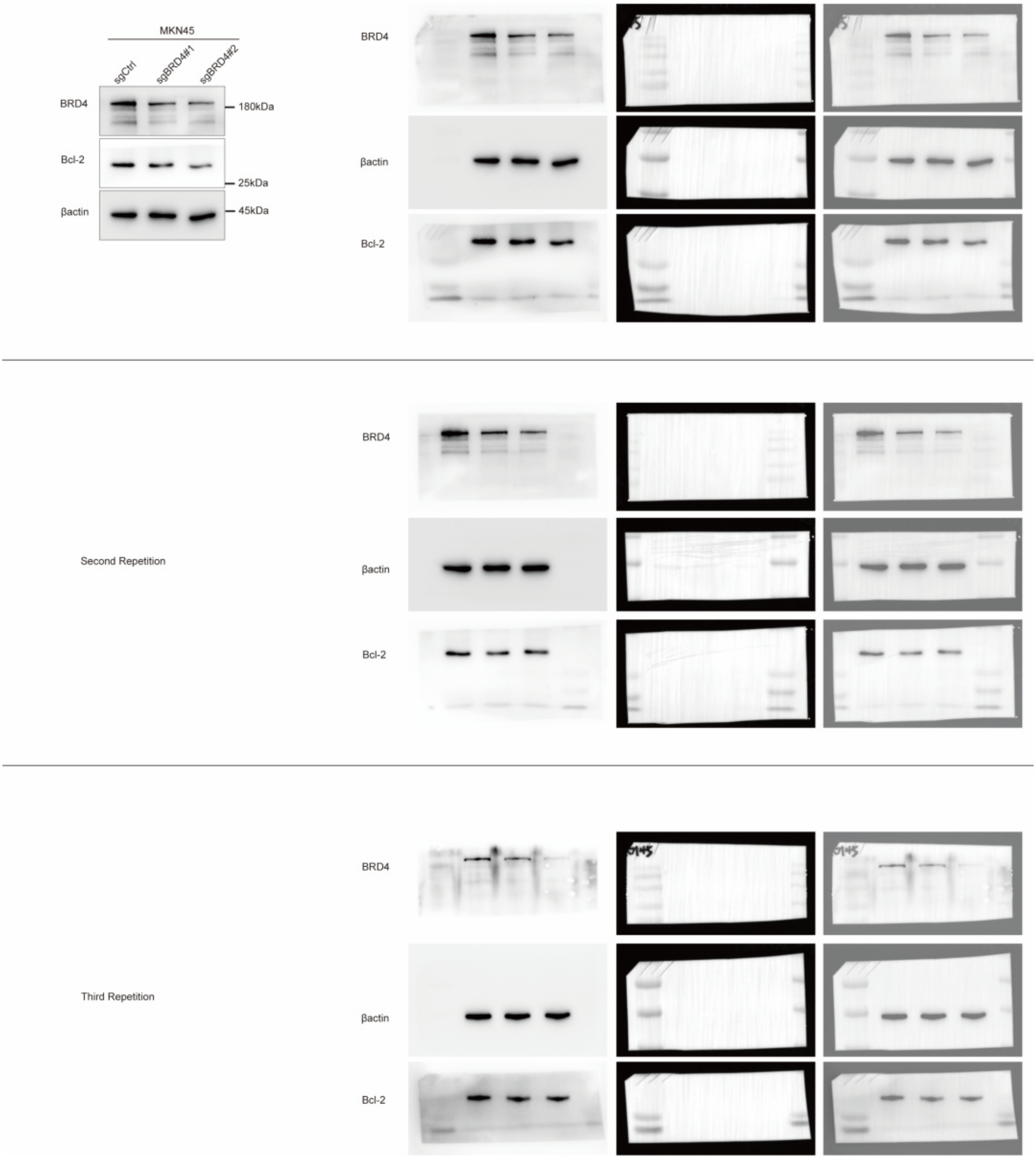
MKN45
sgCtrl
sgBRD4#1
sgBRD4#2
BRD4
180kDa
Bcl-2
25kDa
βactin
45kDa
BRD4
βactin
Bcl-2
Second Repetition
BRD4
βactin
Bcl-2
Third Repetition
BRD4
βactin
Bcl-2

Figure5.A

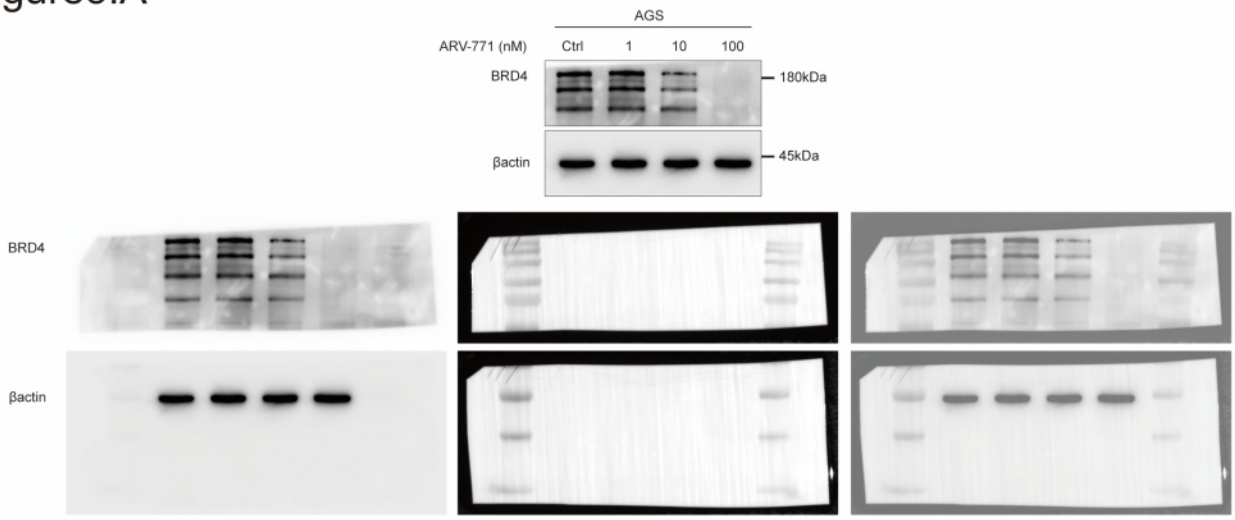

Second Repetition

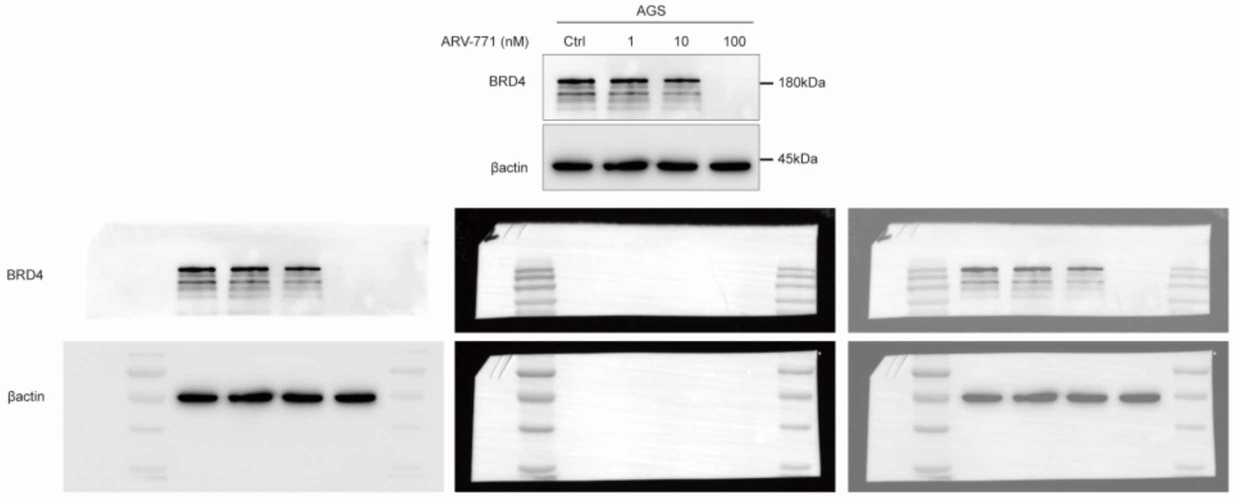

Third Repetition

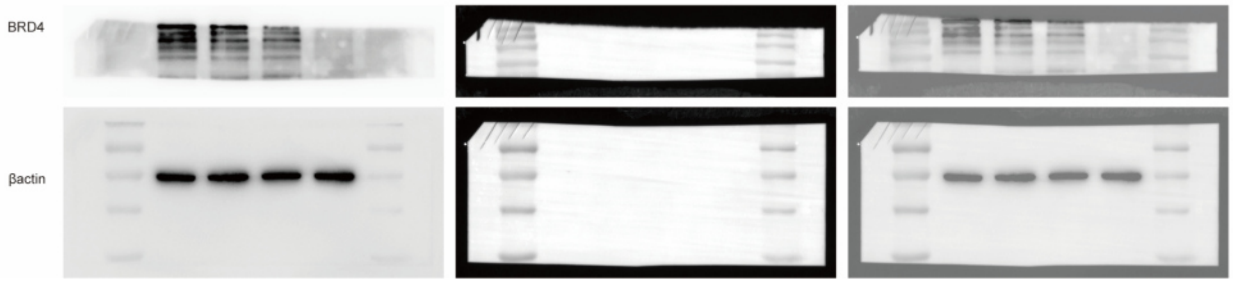

Figure5.E

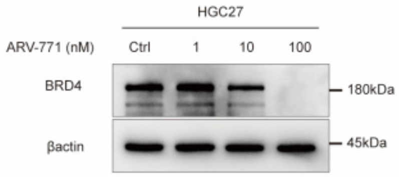

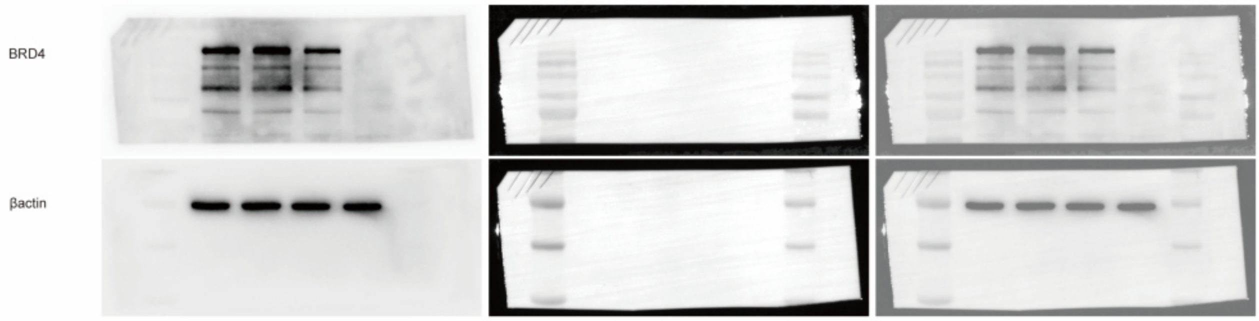

Second Repetition

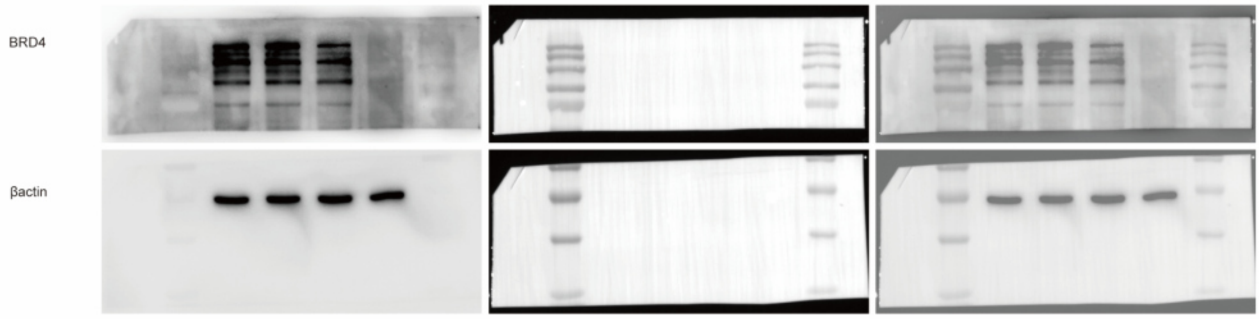

Third Repetition

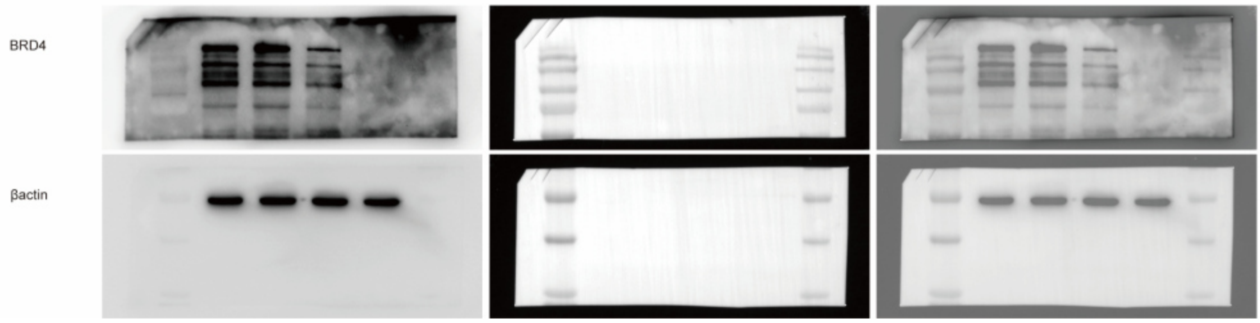

Figure5.I

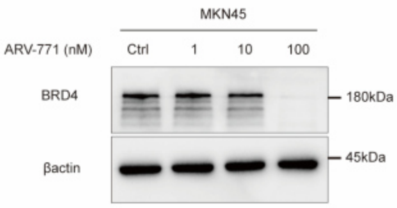

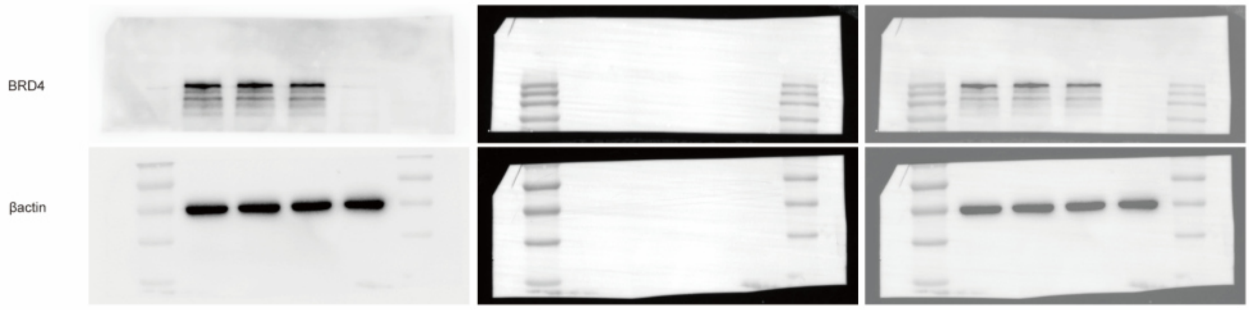

Second Repetition

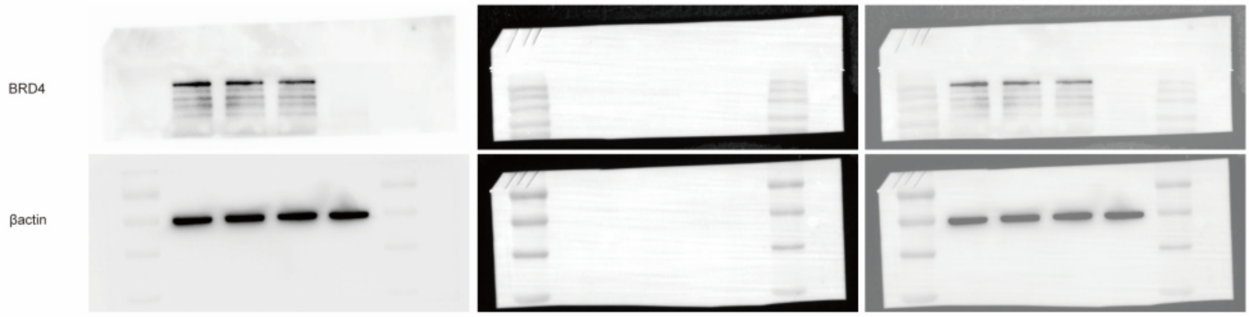

Third Repetition

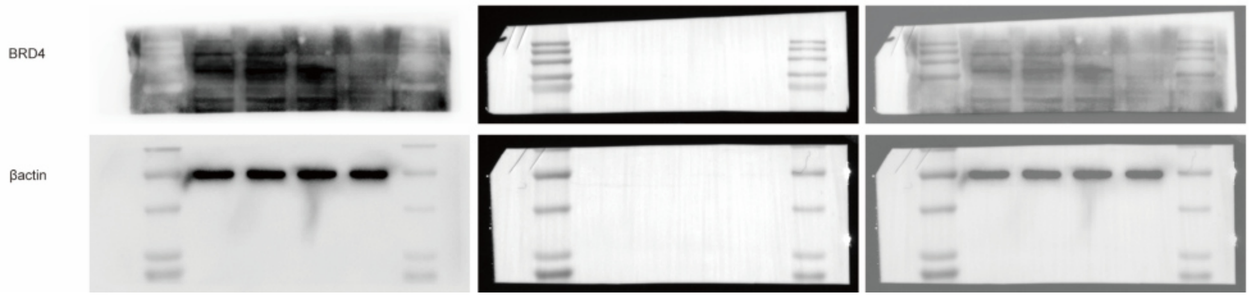

Figure5.M

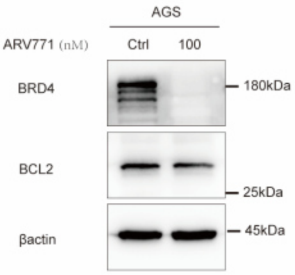

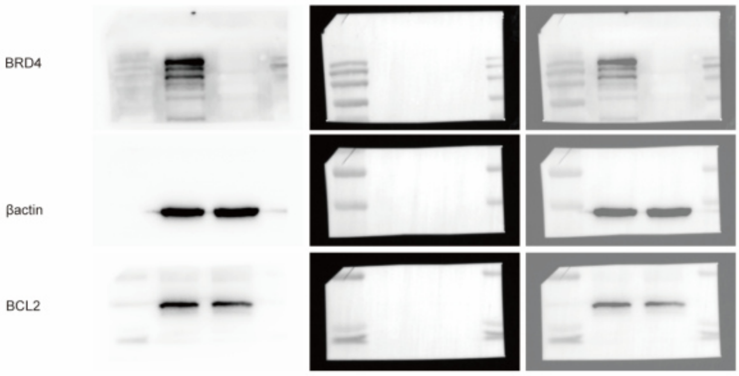

Second Repetition

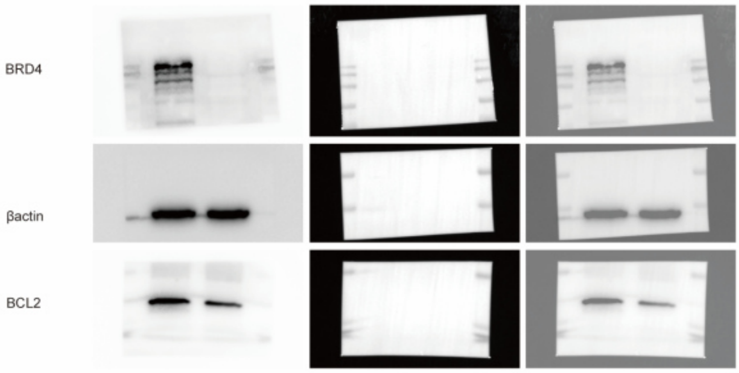

Third Repetition

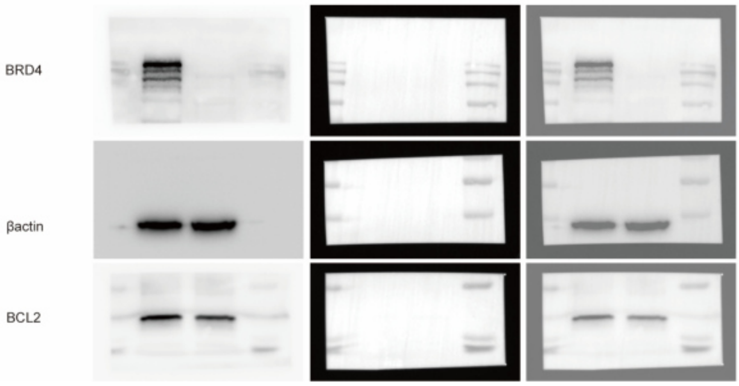

Figure5.N

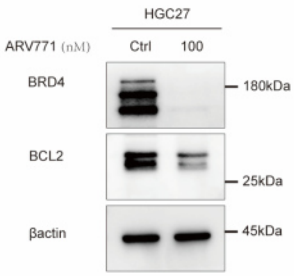

BRD4

βactin

BCL2

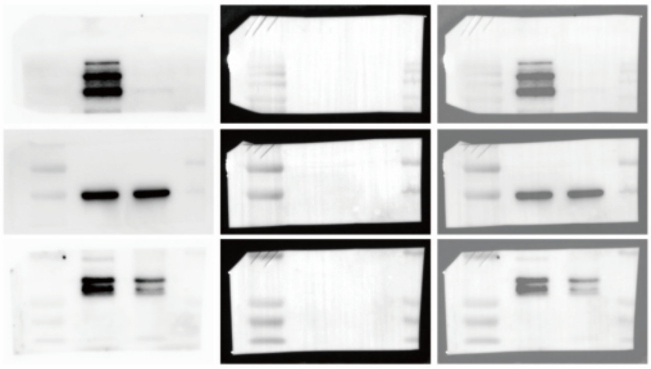

Second Repetition

BRD4

βactin

BCL2

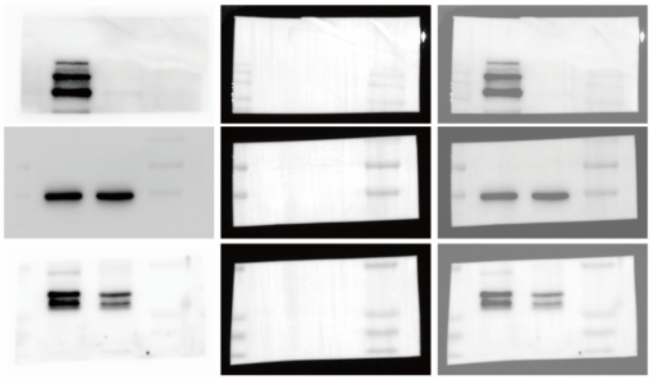

Third Repetition

BRD4

βactin

BCL2

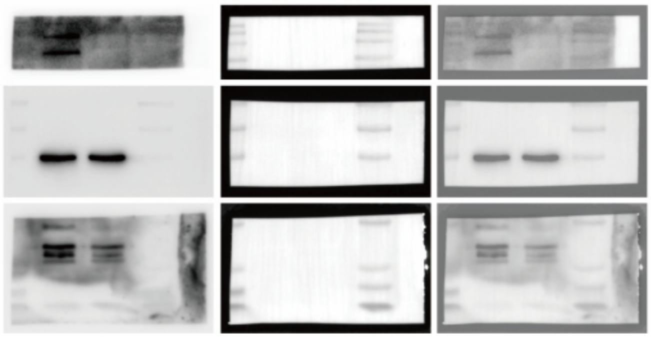

Figure5.O

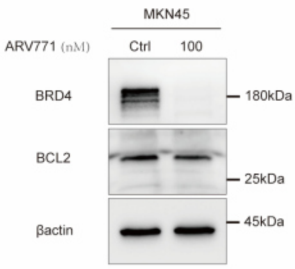

BRD4

βactin

BCL2

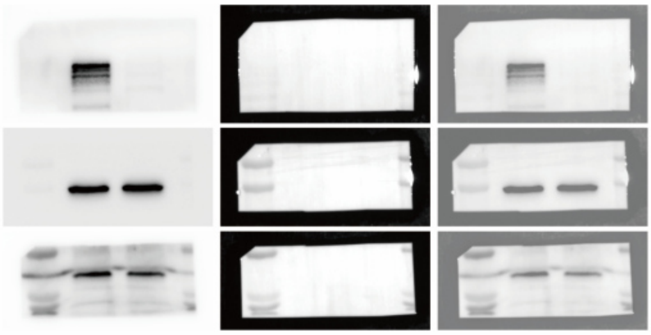

Second Repetition

BRD4

βactin

BCL2

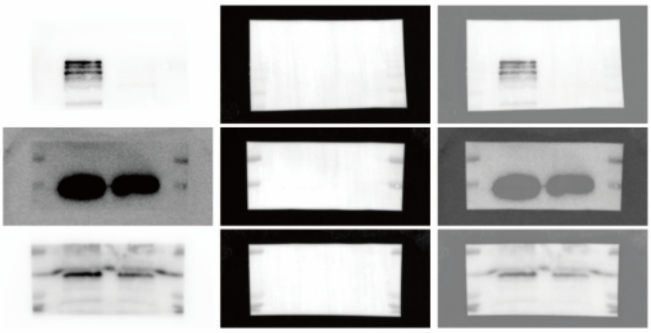

Third Repetition

BRD4

βactin

BCL2

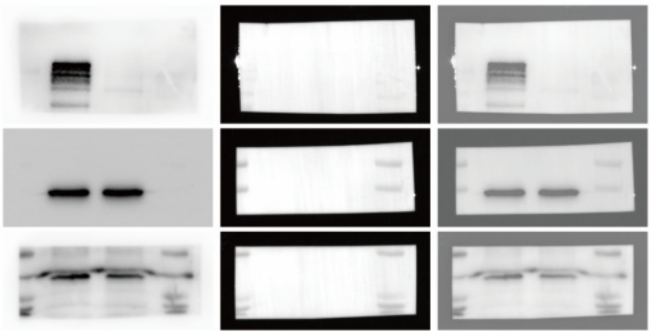

Supplement: Supplementary file 1 [file DataSheet2.pdf]
