# Supplementary figures and images for: Targeting BRD4 in gastric cancer: promoting apoptosis and suppressing tumor progression
Source: Front Pharmacol. 2026 Jun 24;17:1835830. doi: 10.3389/fphar.2026.1835830 (PMC13341472; doi:10.3389/fphar.2026.1835830)

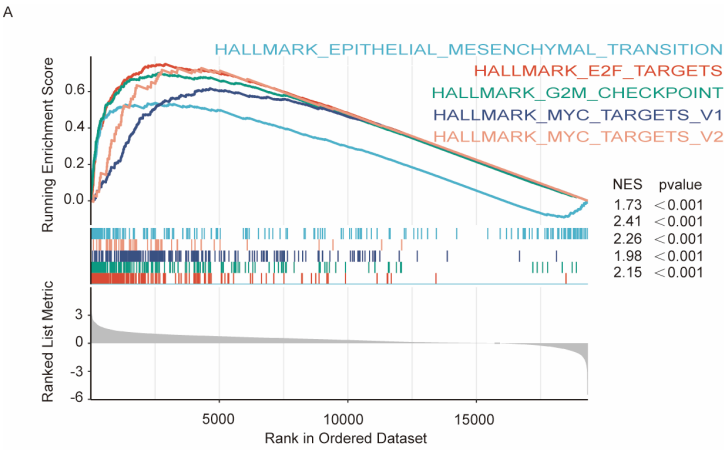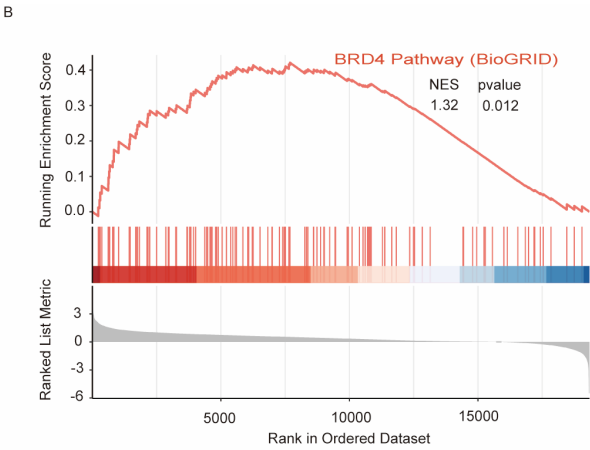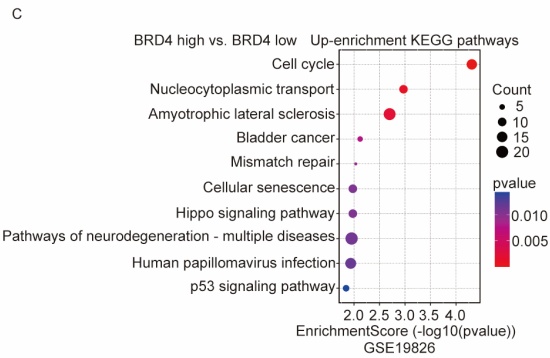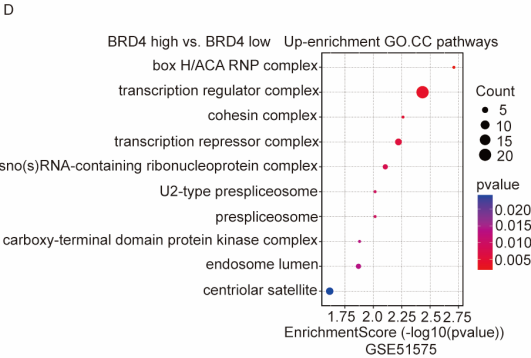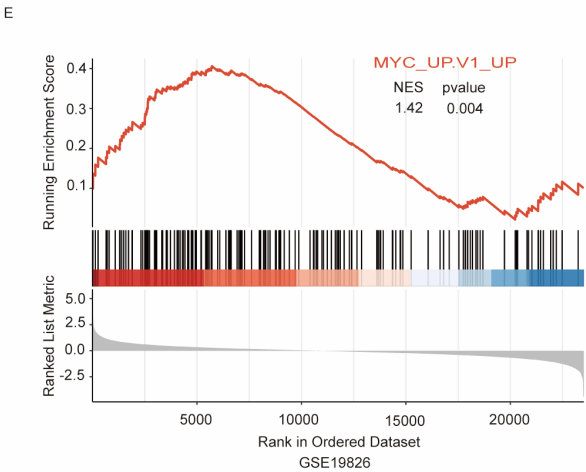

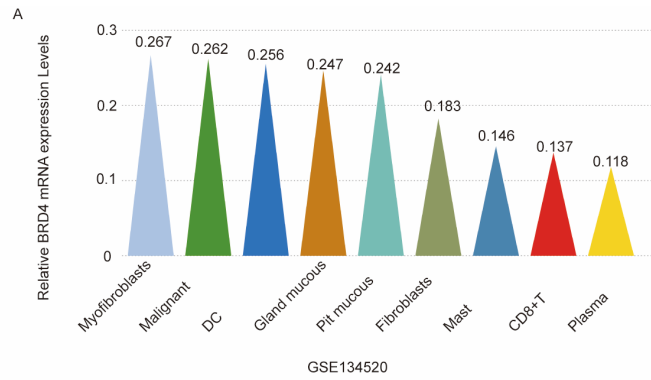

**B**

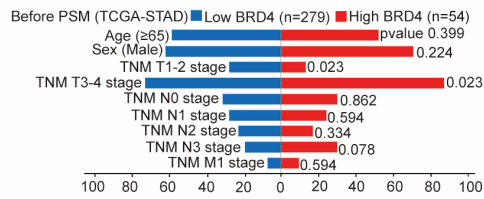

**C**

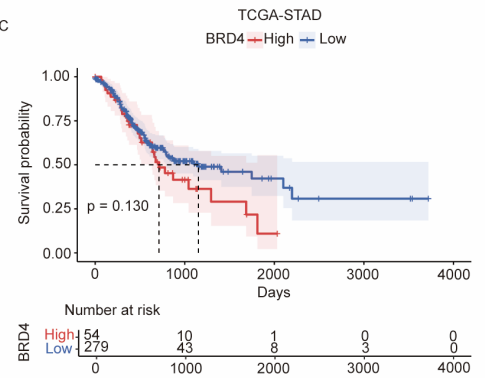

**D**

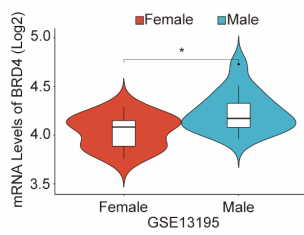

**E**

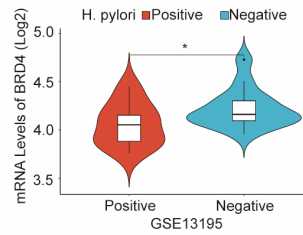

**F**

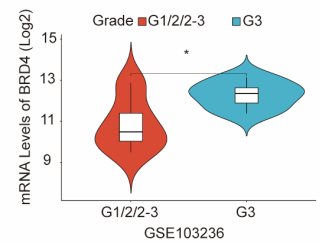

A

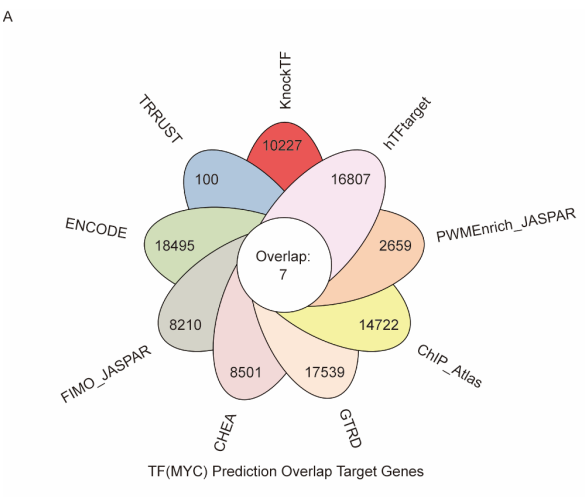

B

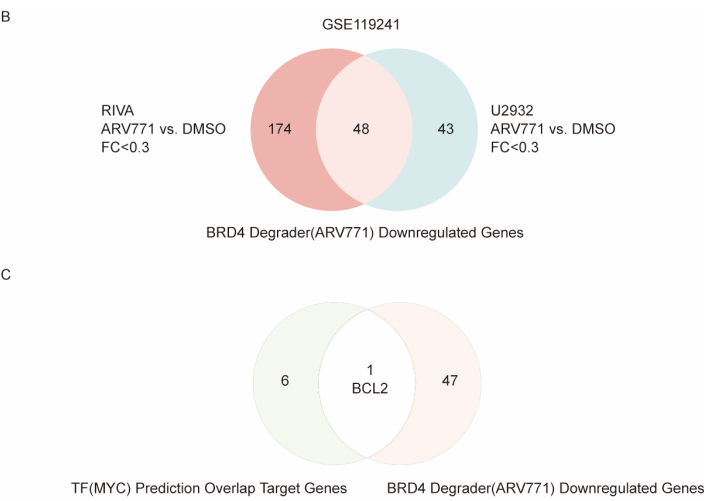

Supplement: Supplementary file 2 [file DataSheet1.pdf]
